# A cytosolic NAD^+^-dependent GPDH from maize (*ZmGPDH1*) is involved in conferring salt and osmotic stress tolerance

**DOI:** 10.1186/s12870-018-1597-6

**Published:** 2019-01-09

**Authors:** Ying Zhao, Meng Liu, Lin He, Xin Li, Feng Wang, Bowei Yan, Jinpeng Wei, Changjiang Zhao, Zuotong Li, Jingyu Xu

**Affiliations:** 1 Key Lab of Modern Agricultural Cultivation and Crop Germplasm Improvement of Heilongjiang Province, Daqing Key Lab of Straw Reclamation Technology Research and Development, College of Agriculture, Heilongjiang Bayi Agricultural University, Daqing, 163319 China; 2 Key Lab of Maize Genetics and Breeding, Heilongjiang Academy of Agricultural Sciences, Harbin, 150000 China

**Keywords:** Glycerol-3-phosphate dehydrogenase, Glycerol, Antioxidants, Redox homeostasis, Salt stress, Osmotic stress, Maize (*Zea mays* L*.*)

## Abstract

**Background:**

Plant glycerol-3-phosphate dehydrogenase (GPDH) catalyzes the reduction of dihydroxyacetone phosphate (DHAP) to produce glycerol-3-phosphate (G-3-P), and plays a key role in glycerolipid metabolism as well as stress responses.

**Results:**

In this study, we report the cloning, enzymatic and physiological characterization of a cytosolic NAD^+^-dependent GPDH from maize. The prokaryotic expression of *ZmGPDH1* in *E.coli* showed that the enzyme encoded by *ZmGPDH1* was capable of catalyzing the reduction of DHAP in the presence of NADH. The functional complementation analysis revealed that *ZmGPDH1* was able to restore the production of glycerol-3-phosphate and glycerol in *AtGPDHc*-deficient mutants. Furthermore, overexpression of *ZmGPDH1* remarkably enhanced the tolerance of *Arabidopsis* to salinity/osmotic stress by enhancing the glycerol production, the antioxidant enzymes activities (SOD, CAT, APX) and by maintaining the cellular redox homeostasis (NADH/NAD^+^, ASA/DHA, GSH/GSSG). *ZmGPDH1* OE *Arabidopsis* plants also exhibited reduced leaf water loss and stomatal aperture under salt and osmotic stresses. Quantitative real-time RT-PCR analyses revealed that overexpression of *ZmGPDH1* promoted the transcripts accumulation of genes involved in cellular redox homeostasis and ROS-scavenging system.

**Conclusions:**

Together, these data suggested that *ZmGPDH1* is involved in conferring salinity and osmotic tolerance in *Arabidopsis* through modulation of glycerol synthesis, stomatal closure, cellular redox and ROS homeostasis.

**Electronic supplementary material:**

The online version of this article (10.1186/s12870-018-1597-6) contains supplementary material, which is available to authorized users.

## Background

It has been shown that glycerol-3-phosphate (G-3-P) serves as a significant intermediary metabolite that connects multiple metabolic pathways, such as gluconeogenes, glycolysis and glycerolipid synthesis [1, 2]. Recent evidences proved that G-3-P also plays a crucial role in adapting to adverse stresses, including salinity, pathogenic microbes, freezing and anaerobic stresses [3]. In higher plants, G-3-P can be biosynthesized through two major pathways. In the first route, G-3-P is generated by NAD^+^-dependent GPDH (EC 1.1.1.8)-mediated reduction of DHAP; while in the second route, G-3-P is produced from glycerol through phosphorylation catalyzed by glycerol kinase (EC 2.7.1.30) [4].

Multiple forms of GPDH have been identified from eukaryotes, and most of them are proved to be key regulators in stress responses [5–7]. There are five GPDH isoforms in *Arabidopsis*, which are associated with different subcellular organelles: one mitochondrial FAD-dependent GPDH (EC 1.1.99.5), two plastidic NAD^+^-dependent GPDHs and two cytosolic NAD^+^-dependent GPDHs [5, 8–10]. Previous studies demonstrated that the *AtGPDHc2* gene encoded a cytosol-targeted GPDH which is involved in pathogen-elicited defense responses in *Arabidopsis* via its effects on the provision of G-3-P [5]. Plants deficient in plastid-localized GPDH (*SFD1*/*GLY1*) exhibited a serious impairment in plastidal glycerolipids pathway of *Arabidopsis* and overexpression of *SFD1*/*GLY1* could increase the plastidic lipid contents as well as the photosynthetic assimilation rate in transgenic rice plants [11].

In addition to their pivotal role in lipid metabolism, plants GPDHs also participate in modulating the intracellular redox status through the mitochondrial G-3-P shuttle system [8, 9]. In *Arabidopsis thaliana*, a mitochondrial FAD-GPDH (EC 1.1.99.5) encoded by the gene *AtGPDHm1*, along with a cytosolic NAD^+^-dependent GPDH (EC 1.1.1.8) encoded by the gene *AtGPDHc1*, was capable of forming the mitochondrial G-3-P shuttle [8]. The operation of G-3-P shuttle is of vital importance to preserve the homeostasis of NADH/NAD^+^ ratio, which is a prerequisite for cells to keep normal metabolic activities. In previous studies, it has been recognized that the expression of *AtGPDHc1* and *AtGPDHm1* is dramatically induced under a variety of stress conditions, like oxygen availability, salinity and dehydration [8, 10]. *AtGPDHc1* knock-out mutants are more sensitive to abscisic acid (ABA) than wild-type (WT) plants, and have failed to stabilize the balance of NADH/NAD^+^ [8]. Loss of *AtGPDHc1* also affected other metabolic pathway involved in redox shuttling, such as mitochondrial malate/OAA shuttle [8].

The characteristics of *GPDH* genes in relation to salinity or osmotic tolerance have been described in some halophilic microalga species [6, 12, 13]. A putative phosphoserine phosphatase (PSP) domain has been found in GPDH isoforms from *Dunaliella salina* (*DsGPDH2*, *G3PDH*) and *Chlamydomonas reinhardtii* (*CrGPD2*), which can serve as glycerol-3-phosphatase (EC 3.1.3.2.1) enzyme and directly catalyze the conversion of DHAP to glycerol under high osmotic environment [14–16]. Furthermore, the transcription of mushroom *GPDH* gene is greatly stimulated by drought and salinity conditions; and overexpression of *PsGPD* improves the salinity tolerance of transgenic rice by increasing the osmotic potential and stomatal conductance [17].

Although the importance of *GPDH* genes in stress responses is well documented in yeast, algae as well as a few plants, there is scarce information about their functions in field crops. Salt and osmotic stresses are the major environmental factors that seriously influence crop growth and productivity [18]. Here, we isolated and characterized a cytosol-localized GPDH (*ZmGPDH1*) gene from maize, which had functional NAD^+^-dependent GPDH activity, and apparent transcriptional response to salinity and mannitol treatments. In addition, overexpression of *ZmGPDH1* in *AtGPDHc*-deficient mutant and WT lines enhanced tolerance of transgenic *Arabidopsis* to salinity and osmotic stresses, with higher glycerol level, lower fluctuation of cellular redox status and stronger ROS antioxidant defense in comparison to both *atgpdhc2* mutant and WT plants. The results showed that *ZmGPDH1* was pivotal in strengthening salt and osmotic stress tolerance by regulating glycerol production, redox homeostasis and ROS antioxidant defense.

## Results

### *ZmGPDH1* encodes a cytosol-targeted protein with NAD^+^-dependent GPDH activity

One GPDH gene was originally obtained through BLAST searching against the maize genome utilizing the reported *AtGPDHc*2 as query [5], the retrieved gene was designated as *ZmGPDH1*. The full length CDS of *ZmGPDH1* was cloned, which had 458 amino acids and an apparent molecular mass of 51 kDa. The complete CDS sequence of *ZmGPDH1* was submitted to GeneBank with the following accession number: MH460963. The sequence alignment revealed that ZmGPDH1 exhibited very high protein sequence identity (77%) to AtGPDHc2, and both proteins consisted of one C-terminal GPD domain (PF07479) that represents DHAP-binding site and one N-terminal NAD-binding domain (PF01210), suggesting that *ZmGPDH1* encodes an NAD^+^-dependent GPDH (Additional file 1: Figure S1).

**Fig. 1:**
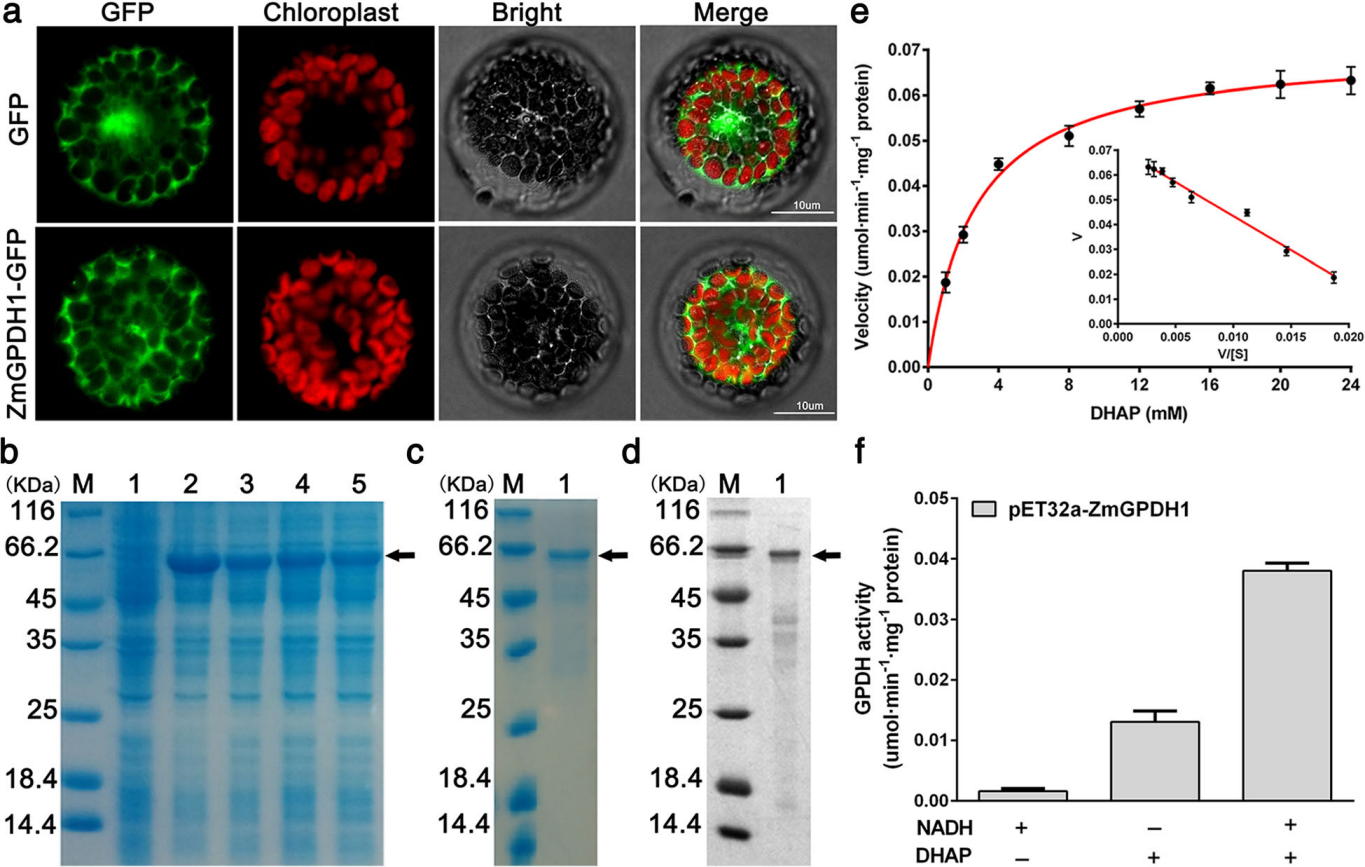
*ZmGPDH1* encodes a cytosol-targeted protein with GPDH activities. **a** Subcellular localization of *ZmGPDH1*. Confocal microscopy observation of *pBI121*-*ZmGPDH1::GFP* or *pBI121*-GFP in transgenic *Arabidopsis* mesophyll protoplasts. Bars = 10 μm. **b** Coomassie-stained 12% SDS-PAGE of cell extract from (Lane 1) Rosetta (DE3) *Escherichia coli* strain and (Lane 2–5) DE3 expressing His-tagged ZmGPDH1 protein. **c** Coomassie-stained 12% SDS-PAGE of (Lane 1) purified ZmGPDH1 fusion protein. **d** Western blot analysis of (Lane 1) purified ZmGPDH1 fusion protein using anti-6 × His antibody as probe. **e** The kinetic properties of ZmGPDH1 with regard to DHAP. **f** GPDH enzyme activities from purified ZmGPDH1 fusion protein. The reaction was performed in the presence (+) and absence (−) of NADH and DHAP, respectively

To certify its subcellular localization, the coding region of *ZmGPDH1* was fused to the N-terminal end of *GFP* reporter gene, and the construct was transformed into wild-type (WT) *Arabidopsis*. The mesophyll protoplasts of *p35S*-*ZmGPDH1::GFP* and *p35S::GFP* (control) transgenic *Arabidopsis* plants were isolated and monitored (Fig. 1a). The free GFP was distributed in cytosol as well as nucleus, whereas the ZmGPDH1-GFP was specifically located in cytosol. Meanwhile, the same result was also found in rice mesophyll protoplasts temporarily expressing the ZmGPDH1-GFP together with the cytosol-marker (mkate protein), which demonstrated that ZmGPDH1 was a cytosol-localized protein (Additional file 2: Figure S2).

To further study the catalytic characteristics of ZmGPDH1, the recombinant protein generated by the *E. coli* Rosetta (DE3) strain expressing plasmid of 6 × His tagged *ZmGPDH1* was purified with a Ni-NTA column. A ZmGPDH1-His fusion protein with an expected size of 65 kDa (consisting of target gene and histidine marker) was identified by SDS/PAGE and Western blot (Fig. 1b, c and d). The recombinant ZmGPDH1 protein was assayed for its kinetic properties relative to the substrate DHAP. Using Eadie-Hofstee plot, the *K*_m_ and *V*_max_ of DHAP were estimated as 2.75 mM and 0.071 umol·min^− 1^·mg^− 1^ protein, respectively (Fig. 1e); besides, addition of NADH strongly stimulated the enzyme activity (Fig. 1f). These results indicated that the purified ZmGPDH1 protein was able to catalyze the reduction of DHAP with the assistant of NADH. Furthermore, the optimum pH of the enzyme activity was determined to be pH 7.0 and the optimum temperature was 35 °C, respectively (Additional file 3: Figure S3).

### Expression profiles of *ZmGPDH1* in response to NaCl or mannitol treatment

**Fig. 2:**
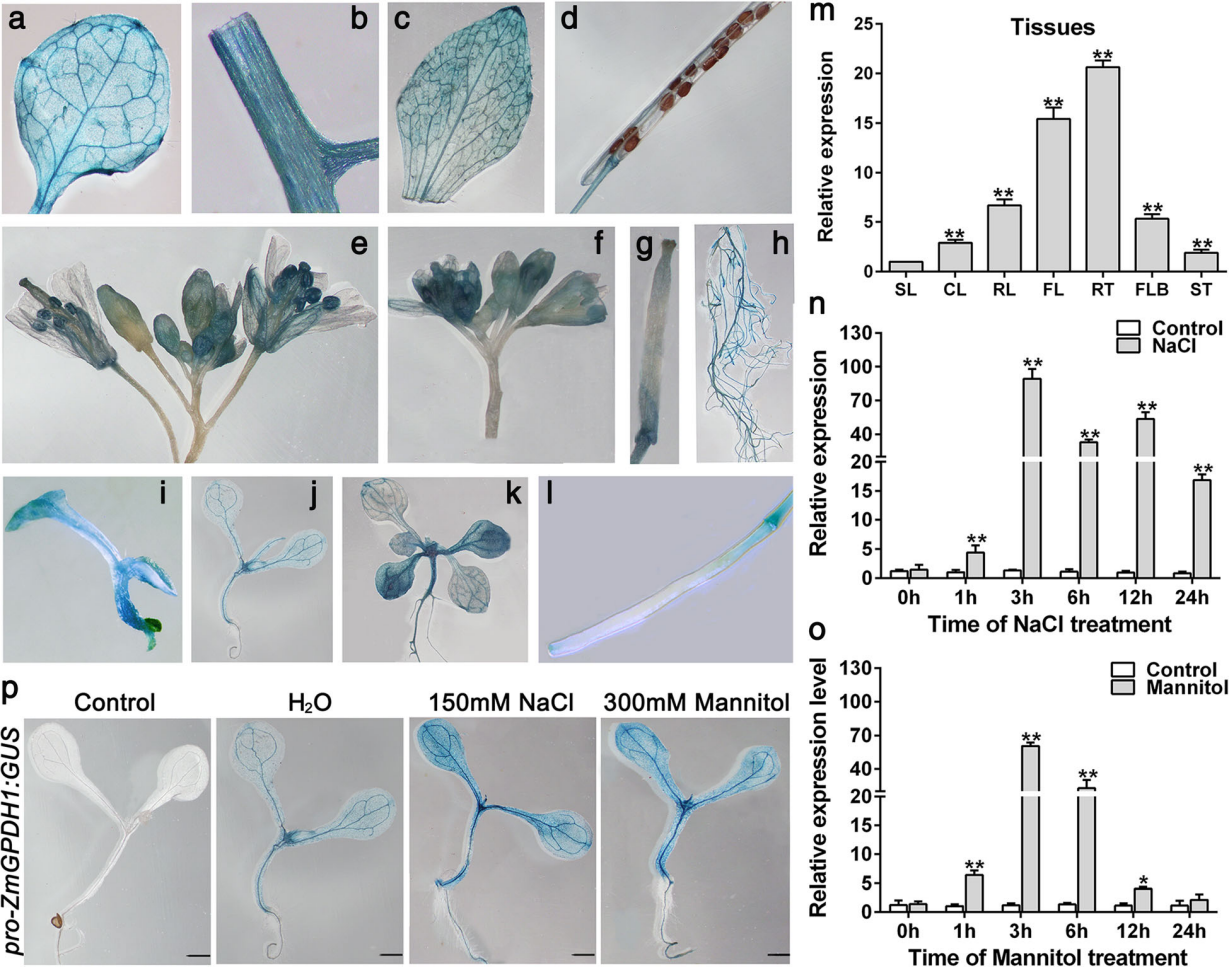
Expression characteristics of *ZmGPDH1* gene. *ZmGPDH1* promoter-GUS assay in various tissues, including (**a**) rosette leaf, (**b**) stem, (**c**) cauline leaf, (**d**) mature silique, (**e**) flower, (**f**) flower bud, (**g**) stigma, (**h**) root, (**i**) 4-day-old plant, (**j**) 7-day-old plant, (**k**) 14-day-old plant and (**l**) immature silique. (**m**) The transcript level of *GUS* gene in different tissues of 5-week-old *proZmGPDH1*::GUS transgenic plants (T3 generation), including stems (ST), rosette leaves (RL), cauline leaves (CL), roots (RT), flowers (FL), flower buds (FLB), siliques (SL). The expression of *GUS* gene in SL was used as a calibrator. The transcriptional response of *ZmGPDH1* in maize roots exposed to (**n**) NaCl or (**o**) mannitol treatments. The expression of *ZmGPDH1* in untreated samples (control) harvested at each time point was used as a calibrator. (**p**) The analysis of GUS activity in response to mannitol or NaCl treatment. 7-day-old *proZmGPDH1*::GUS transgenic seedlings were transferred to either half-strength MS plates (1/2 MS), plates with 300 mM mannitol or plates with 150 mM NaCl for 12 h before GUS staining. Non-transformed wild-type (WT) was used as a control. Bars = 100 μm. The asterisks represented a significant difference as determined by the Student′s t-test (*P < 0.05, ** *P* < 0.01)

To examine the potential functions of *ZmGPDH1* in response to plant growth, the promoter region of *ZmGPDH1* was amplified and fused to N-terminus of *GUS* reporter gene, and the *proZmGPDH1::GUS* construct was transformed to WT *Arabidopsis* plants. In 4-day-old transgenic seedlings, high GUS activity was observed in shoots apical meristem; in 7-day-old or 14-day-old seedlings, high GUS activity was observed in young leaves and petioles (Fig. 2). In 5-week-old *proZmGPDH1*::GUS transgenic plants, strong constitutive *GUS* activity was shown in flowers, roots, rosette leaves, and stems. To further verify these findings, quantitative real-time RT-PCR (qRT-PCR) was carried out, and the level of *GUS* transcripts in siliques of 5-week-old *proZmGPDH1::GUS* transgenic plants was used as calibrator (Fig. 2m). Consistent with the result of *GUS* activity assay, the *GUS* transcripts could be observed in all tissues examined, with high levels of transcription in roots, flowers and rosette leaves. These results suggested that the *ZmGPDH1* promoter exhibited a tissue-specific expression pattern.

It has been proven that *GPDH* genes are essential for stress adaptations in *yeast*, marine algae and *Arabidopsis* [5, 17–19]. Therefore, to explore its possible involvement in stress responses in maize, we first analyzed the transcripts accumulation of *ZmGPDH1* under different stress treatments. The qRT-PCR results showed that *ZmGPDH1* was remarkably up-regulated by both salinity and osmotic stresses in maize roots, which reached the highest level at 3 h under both treatments (Fig. 2n and o). Notably, the expression of *proZmGPDH1::GUS* was also enhanced in the presence of NaCl and mannitol treatments (Fig. 2p), suggested that *ZmGPDH1* was involved in the transcriptional response during salt or osmotic adaptions.

### Overexpression of *ZmGPDH1* enhanced the tolerance of *Arabidopsis* to salt and osmotic stresses

**Fig. 3:**
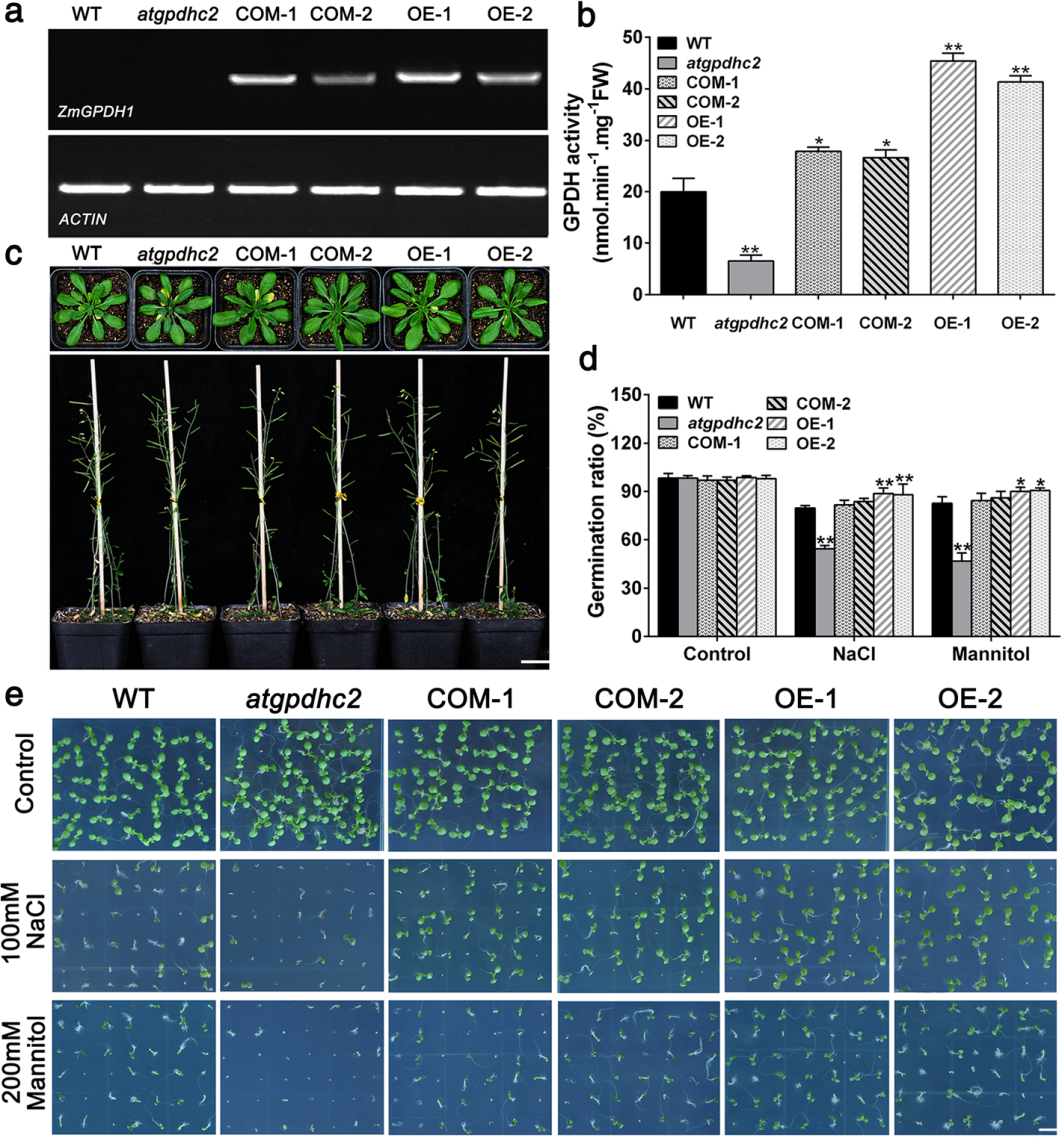
Germination characteristics of the *ZmGPDH1* transgenic lines in response to salt or osmotic stresses. **a** Reverse transcription PCR (RT-PCR) of *ZmGPDH1* transcripts in the WT, *atgpdhc2*, *ZmGPDH1* transformed *atgpdhc2* (COM-1, COM-2) and transgenic WT (OE-1, OE-2) plants. *ACTIN* served as the internal reference. **b** The GPDH activities in *ZmGPDH1* transgenic lines compared with the WT and *atgpdhc2*. **c** Morphological comparison among the WT, *atgpdhc2*, COM-1, COM-2, OE-1 and OE-2 lines grown in soil. Top panel, 28-day-old plants; bottom panel, 56-day-old plants. Bars = 200 mm. **d** The germination rate of the six lines under normal (control), NaCl or mannitol conditions at day 5 after imbibition. **e** Images of seeds germinated on either half-strength MS medium (control), medium with 100 mM NaCl or medium with 200 mM mannitol for 7 days. Bars = 50 mm. Asterisks indicated significant differences from the WT, as determined by Student′s t-test (*P < 0.05, ** *P* < 0.01)

Next, to further understand how *ZmGPDH1* responds to salt or osmotic stress, *ZmGPDH1* transformed *AtGPDHc2*-deficient mutant (COM-1, COM-2) and WT (OE-1, OE-2) lines were generated. The T-DNA insertion mutant of *AtGPDHc2* was identified by PCR and reverse-transcription PCR (RT-PCR), and the homozygous *atgpdhc2* mutants were selected for the transformation studies (Additional file 4: Figure S4). The RT-PCR results showed that the full-length transcription product of *ZmGPDH1* was absent in the WT *Arabidopsis* or *atgpdhc2* mutant, but existed in all *ZmGPDH1* transgenic lines (Fig. 3a). The enzymatic assay revealed that GPDH activities of *ZmGPDH1* overexpression lines (OE-1, OE-2) were 2.1- to 2.3-fold higher than that in the WT, while GPDH activities of *atgpdhc2* mutant was 31% of that in the WT. Meanwhile, the GPDH activities of *ZmGPDH1* complementation lines (COM-1, COM-2) were 1.2- to 1.3-fold higher than that in the WT, which indicated that *ZmGPDH1* was successfully expressed and functioned with GPDH activity in both OE and COM lines (Fig. 3b). Compared with WT, *atgpdhc2,* COM and OE lines showed no aberrant phenotype under normal growth conditions, no matter in the vegetative growth phase or reproductive developmental stages (Fig. 3c).

Additionally, to evaluate the performance of *ZmGPDH1* transgenic lines in response to salt or osmotic stresses, the seeds of WT, *atgpdhc2,* COM and OE lines were germinated on half-strength MS mediums containing 100 mM NaCl or 200 mM mannitol. As shown in Fig. 3d and e, seed germination of *atgpdhc2* was severely delayed in comparison to the WT, whereas the germination rate of *ZmGPDH1* OE seeds was much higher than that of other lines. The *ZmGPDH1* COM seeds displayed stress-sensitive morphologies similarity to the WT, albeit they had a relative higher germination rate (Fig. 3d and e).

**Fig. 4:**
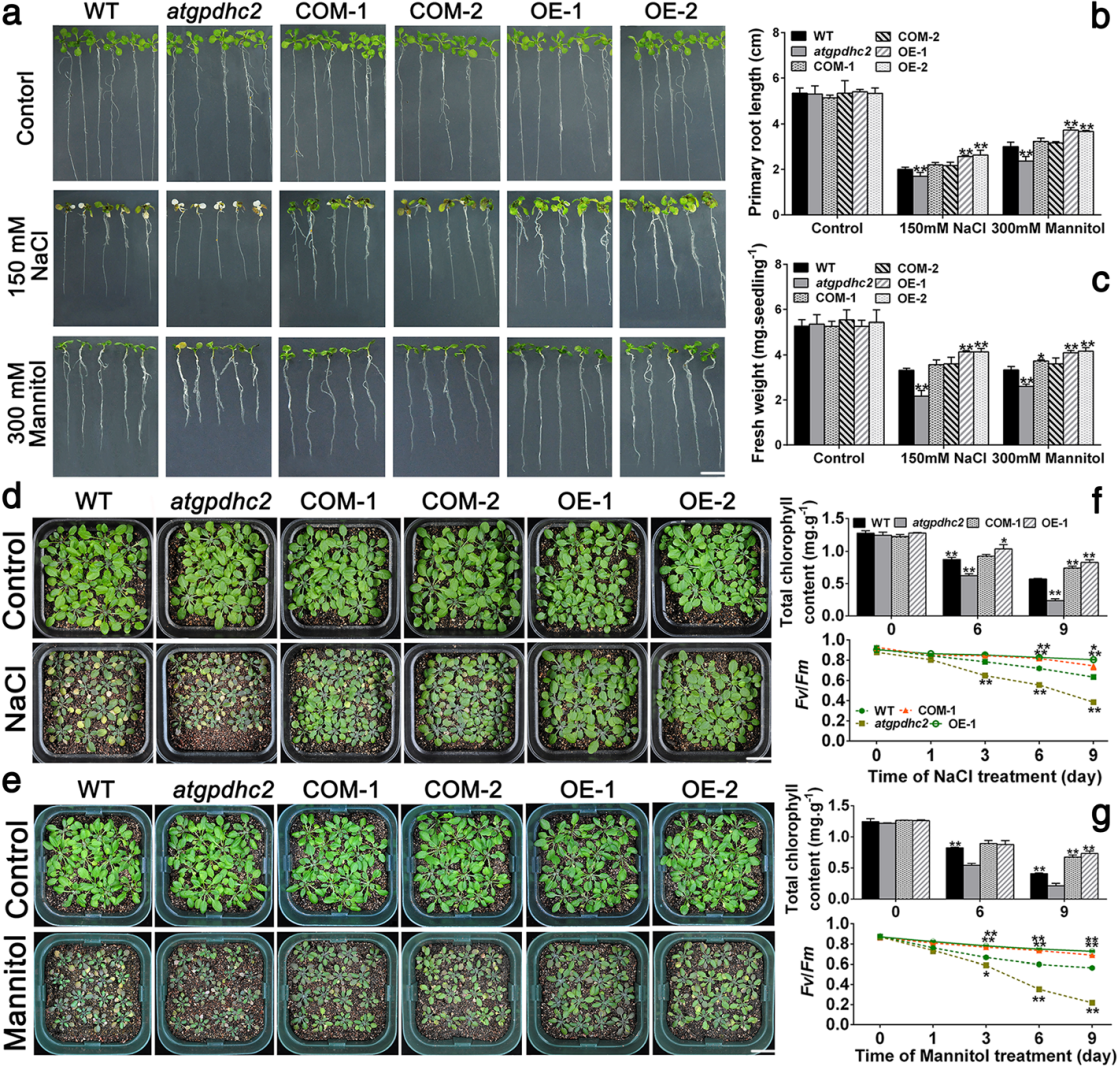
Overexpression of *ZmGPDH1* enhances the salt- and drought-tolerance in transgenic *Arabidopsis*. **a** Images of 7-day-old WT, *atgpdhc2*, complementation (COM-1, COM-2) and overexpression (OE-1, OE-2) plants grown on either half-strength MS plates (control), plates with 300 mM mannitol or 150 mM NaCl for 7 days. Bars = 100 mm. **b** and **c** The primary root length and fresh weight of the six lines after 7 days of treatments. Images of 3-week-old plants irrigated with water (control), 200 mM NaCl (**d**) or 400 mM mannitol (**e**) every 3 days for 9 days. Bars = 200 mm. The total chlorophyll contents and chlorophyll fluorescence (*F*_*v*_/*F*_*m*_) of 3-week-old plants irrigated with water (control), 200 mM NaCl (**f**) or 400 mM mannitol (**g**) over 9 days. Asterisks indicated significant differences (*P < 0.05, ** *P* < 0.01) from the WT, as determined by Student′s t-test

To investigate the impact of *ZmGPDH1* overexpression on salt/osmotic resistance at early seedling stage, 7-day-old WT, *atgpdhc2,* COM and OE lines were transplanted to half-strength MS plates supplemented with 300 mM mannitol or 150 mM NaCl for 7 days. As shown in Fig. 4a, the *atgpdhc2* seedlings displayed much severer stress-hypersensitive phenotype than the WT, with a partial leaf bleaching phenomenon. However, *ZmGPDH1* transformed *atgpdhc2* (COM-1, COM-2) plants showed wild-type-like phenotype under NaCl and mannitol treatments, indicating that overexpression of *ZmGPDH1* could rescue salt and osmotic sensitivity of *atgpdhc2* mutants. In addition, *ZmGPDH1* OE plants exhibited an enhanced tolerance to salt/osmotic stress, and a resulting higher fresh weight and root length compared with the WT (Fig. 4 b and c).

Likewise, when the 3-week-old *Arabidopsis* plants were subjected to 400 mM mannitol or 200 mM NaCl treatment for 9 days, the growth of *atgpdhc2* mutants was strongly inhibited compared with the WT (Fig. 4d and e). Conversely, the *ZmGPDH1* OE or COM plants showed obviously improved tolerance to salinity or osmotic stress relative to the other lines (Fig. 4d and e). Synchronously, the chlorophyll fluorescence parameter (*F*_*v*_/*F*_*m*_) and total chlorophyll content were analyzed in *atgpdhc2*, COM-1, WT and OE-1 plants. Under standard conditions, chlorophyll content and *F*_*v*_/*F*_*m*_ had no differences among all four lines (Day 0); however, after exposure to 400 mM mannitol or 200 mM NaCl treatment, *atgpdhc2* mutant demonstrated a remarkable reduction in *F*_*v*_/*F*_*m*_ and chlorophyll content, whereas the *F*_*v*_/*F*_*m*_ ratio and total chlorophyll content in OE-1 and COM-1 were much higher than that in WT plants (Fig. 4 f and g). These data suggested that overexpression of *ZmGPDH1* helped to enhance the photochemical efficiency in transgenic *Arabidopsis*.

### *ZmGPDH1* regulates glycerol-3-phosphate and glycerol levels under salt and osmotic stresses

**Fig. 5:**
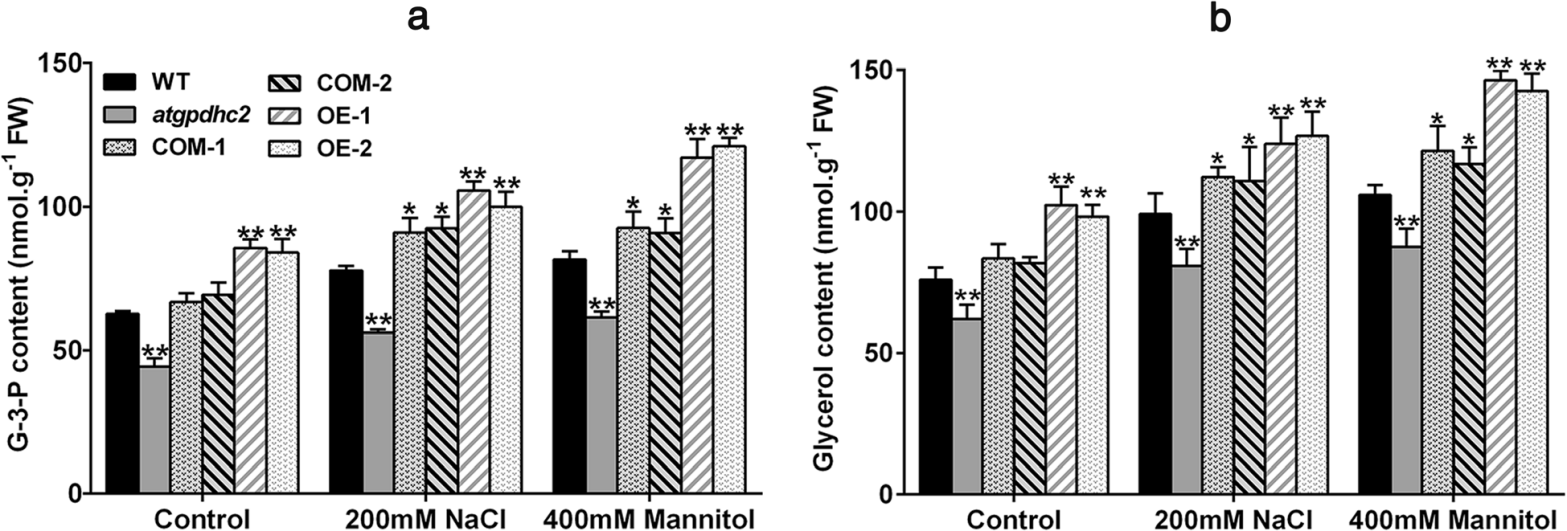
Overexpression of *ZmGPDH1* increases the glycerol-3-phosphate and glycerol levels under salt and osmotic stresses. **a** and **b** The glycerol-3-phosphate and glycerol contents in 3-week-old WT, *atgpdhc2*, COM-1, COM-2, OE-1 and OE-2 seedlings irrigated with water (control), 400 mM mannitol and 200 mM NaCl every 3 days for 6 days. Asterisks indicated significant differences from the WT by Student′s t-test (*P < 0.05, ** *P* < 0.01)

Glycerol is an important compatible solute and the physiological significance of glycerol biosynthesis under salinity or osmotic condition has been reported in many species [4, 12, 20]. G-3-P is a primary substrate for glycerol synthesis [21]. To characterize the effect of *ZmGPDH1* on glycerol metabolism, we assayed G-3-P and glycerol levels in 3-week-old WT, *atgpdhc2*, COM and OE *Arabidopsis* lines treated with 200 mM NaCl or 400 mM mannitol for 6 days. Under normal soil conditions, significant reduction in G-3-P and glycerol contents were observed in *gpdhc2* mutants, while the average contents of G-3-P and glycerol were increased in the OE and COM lines compared with WT *Arabidopsis* (Fig. 5 a and b). After treatment with NaCl or mannitol, the OE and COM plants accumulated higher level of G-3-P and glycerol than the other lines (Fig. 5a and b), indicating that the overexpression of *ZmGPDH1* could promote the cellular glycerol biosynthesis under high salinity or hyperosmotic stress.

### *ZmGPDH1* is essential for redox homeostasis under salt and osmotic stresses

**Fig. 6:**
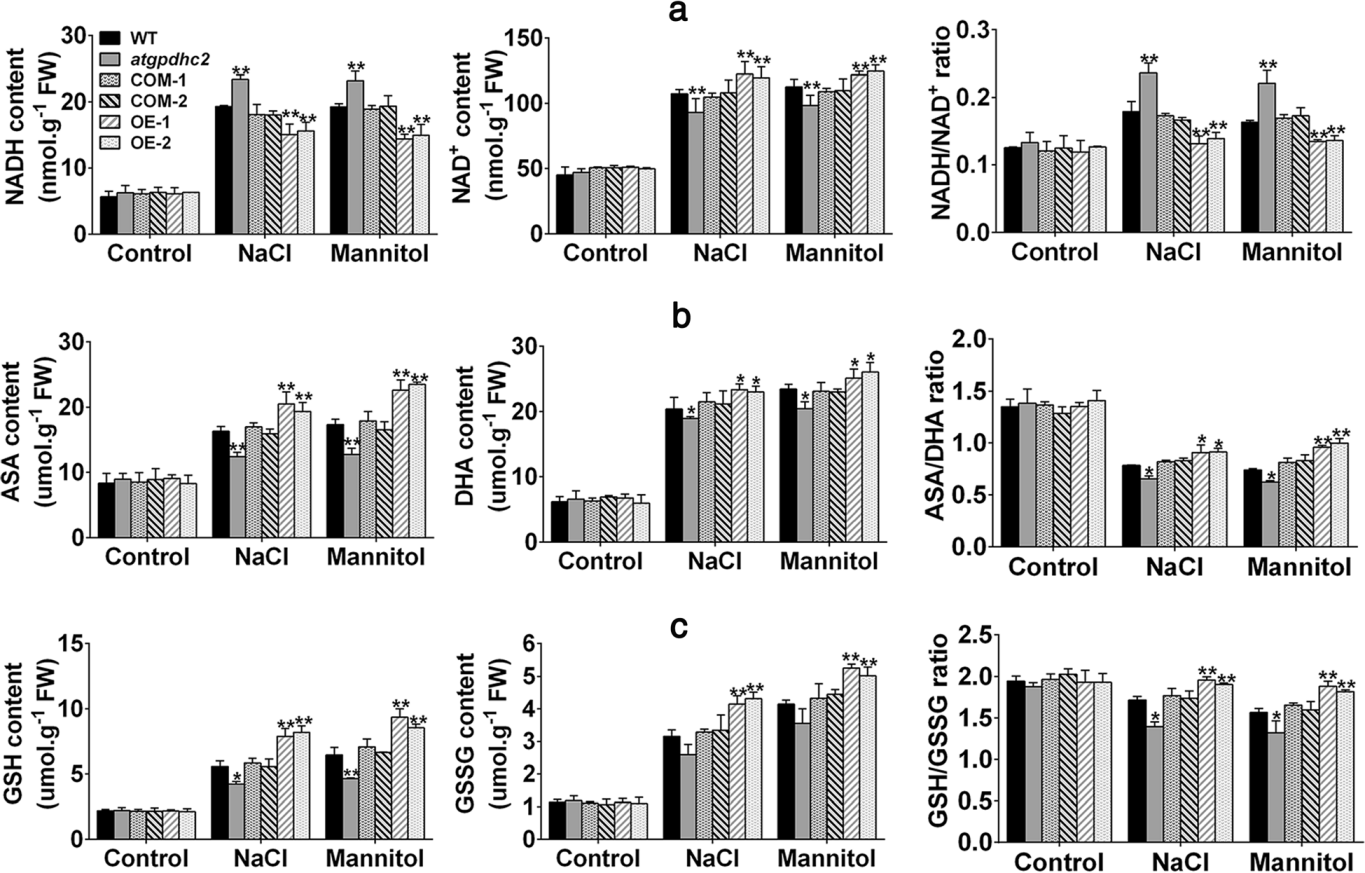
Overexpression of *ZmGPDH1* maintains the redox homeostasis under salt and osmotic stresses. **a** NADH contents, NAD^+^ contents, NADH/NAD^+^ ratios; **b** ASA contents, DHA contents, ASA/DHA ratios; **c** GSH contents, GSSG contents, GSH/GSSG ratio in 3-week-old WT, *atgpdhc2*, COM-1, COM-2, OE-1 and OE-2 seedlings irrigated with water (control), 400 mM mannitol and 200 mM NaCl every 3 days for 6 days. Asterisks indicated significant differences from the WT by Student′s t-test (*P < 0.05, ** *P* < 0.01)

It has been reported that the cytosolic NAD^+^-GPDH is involved in NADH:NAD^+^ recycling by catalyzing the conversion of DHAP to G-3-P using NADH as a reducing equivalent [8]. To validate if overexpression of *ZmGPDH1* could affect NADH/NAD^+^ homeostasis upon salt and osmotic stresses, the fluctuation in redox status of NADH was monitored. Under normal growth condition, no significant differences were observed in NADH, NAD^+^ contents and NADH/NAD^+^ ratio among WT, *atgpdhc2*, COM-1, COM-2, OE-1 and OE-2 lines (Fig. 6a). However, a severe interference in NADH/NAD^+^ homeostasis appeared in all the six lines under salt or mannitol treatment, differences could also be seen in the individual NADH or NAD^+^ contents. Although stress treatments elevated the NADH level in each line, the *ZmGPDH1* OE plants accumulated comparatively lower NADH and higher NAD^+^ contents in comparison to WT plants, resulting in a decreased NADH/NAD^+^ ratio (Fig. 6a). Nevertheless, *atgpdhc2* mutants accumulated more NADH and substantially less NAD^+^ content than WT or COM plants, leading to a higher NADH/NAD^+^ ratio. These results illustrated that overexpression of *ZmGPDH1* might facilitate the oxidation of the excessive reductant (NADH) induced by salt and osmotic stresses, thus increased the NAD^+^/NADH ratio.

To further examine whether overexpression of *ZmGPDH1* could influence the other redox couples, the cellular oxidized and reduced pools of ASA and GSH were also determined. Similarly, the contents of ASA, GSH and their oxidized form DHA, GSSG did not change among the six lines under normal growing conditions (Fig. 6b and c). However, when the plants were treated with NaCl or mannitol, the *ZmGPDH1* OE lines maintained relative higher ASA and GSH contents compared with the WT, leading to an even higher ASA/DHA or GSH/GSSG ratio. By contrast, there was a noticeable decline in reduced ascorbate or glutathione as well as the redox ratio (ASA/DHA, GSH/GSSG) in *atgpdhc2* mutants relative to WT or COM lines (Fig. 6b and c). Collectively, these results implied that the founction of *ZmGPDH1* in salinity and osmotic tolerance could partly attribute to sustaining the cellular redox homeostasis.

### *ZmGPDH1* regulates the ROS level and cell death under salt and osmotic stresses

**Fig. 7:**
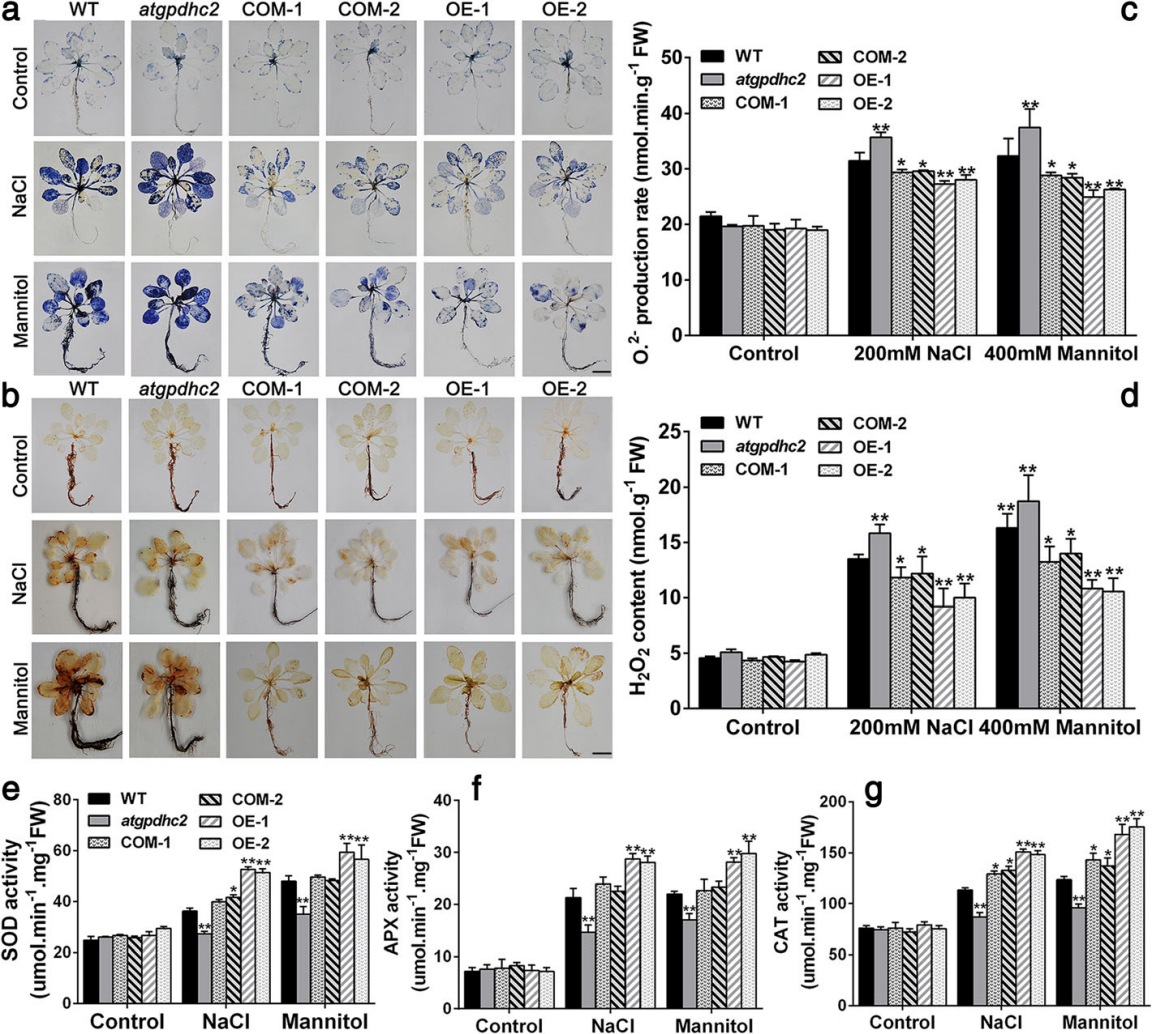
Overexpression of *ZmGPDH1* enhances the ROS scavenging capacity under salt and osmotic stresses. Photographs showing representative (**a**) NBT and (**b**) 3,3-diaminobenzidine (DAB) staining of 3-week-old WT, *atgpdhc2*, COM-1, COM-2, OE-1 and OE-2 plants treated with water (control), 200 mM NaCl and 400 mM mannitol for 2 h. Bars = 50 mm. O_2_^.-^ (**c**) and H_2_O_2_ (**d**) levels in the six lines after 6 days of water (control), 200 mM NaCl or 400 mM mannitol treatment. Enzyme activity of **(e)** superoxide dismutase (SOD), (**f**) catalase (CAT) and (**g**) ascorbate peroxidase (APX) in the six lines treated as above. Asterisks indicated significant differences from the WT by Student′s t-test (*P < 0.05, ** *P* < 0.01)

In plant stress reactions, the redox state is highly correlated with the cellular ROS producing and processing [22, 23]. Hence, the remarkable changes in redox ratios (NADH/NAD^+^, ASA/DHA, GSH/GSSG) in *ZmGPDH1* transformed plants promoted us to investigate the ROS level under salt and osmotic stresses. The Fig. 7a depicted NBT staining of O_2_^.-^, while the Fig. 7b illustrated DAB staining of H_2_O_2_. In both cases, heavier coloration, reflecting the elevated level of ROS, was detected in *atgpdhc2* mutants after NaCl or mannitol treatment. On the contrary, the slighter coloration of NBT and DAB staining were observed in both COM and OE plants under identical conditions. Meanwhile, quantitative measurements showed that the contents of H_2_O_2_ and O_2_^.-^ in COM and OE plants were markedly lower than that in the WT and *atgpdhc2* mutants, suggesting the vital role of *ZmGPDH1* in modulating the cellular ROS accumulation under salt or osmotic stress (Fig. 7c and d). The increased ROS production had the potential to trigger the compensatory responses of antioxidant enzymes, therefore, the activities of ROS-scavenging enzymes, including catalase (CAT), ascorbate peroxidase (APX) and superoxide dismutase (SOD) were also determined in this study [23]. As expected, an enhanced activity of these antioxidant enzymes was detected in all the lines under salinity or osmotic condition, while the elevation in OE or COM lines was more prominent than that in the WT (Fig. 7e, f and g). In reverse, the *atgpdhc2* mutant possessed relatively lower antioxidant enzymes activities compared with the WT.

**Fig. 8:**
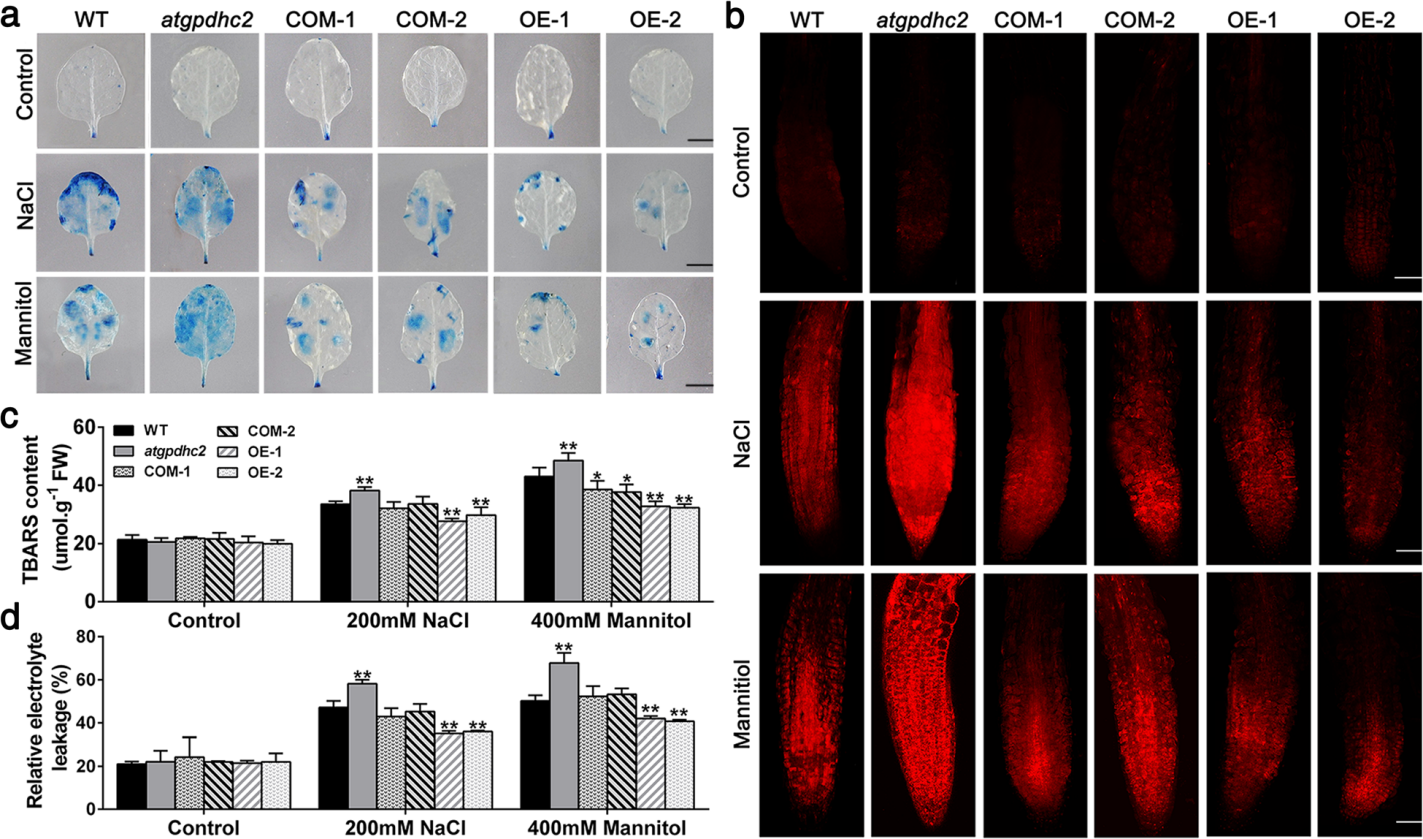
Overexpression of *ZmGPDH1* alleviates the stress-induced membrane injury and cell death. **a** The Evan′s Blue staining in 3-week-old WT, *atgpdhc2*, COM-1, COM-2, OE-1 and OE-2 lines irrigated with water (control), 400 mM mannitol and 200 mM NaCl for 2 h. Bars = 50 mm. **b** The propidium iodide (PI) fluorescence staining in root tips of 7-day-old plants treated on either half-strength MS plates (control), plates with 300 mM mannitol or 150 mM NaCl for 12 h. The images were obtained by cofocal microscope. Bars = 100 μm. TBARS contents (**c**) and the relative electrolyte leakage (**d**) of the six lines after 6 days of water (control), 200 mM NaCl or 400 mM mannitol treatments. Asterisks indicated significant differences from the WT by Student′s t-test (*P < 0.05, ** *P* < 0.01)

Additionally, the oxidative stress mediated cell death was also detected by Evan′s blue and propidium iodide (PI) staining [24]. As shown in Fig. 8a and b, mild intensities of Evan′s Blue and PI staining was observed in all the six lines under the normal conditions. However, the cell death was strongly stimulated in *atgpdhc2* mutants, moderately stimulated in WT or COM and mildly stimulated in OE line after treatment with NaCl or mannitol (Fig. 8a and b). The TBARS and electrolyte leakage levels, as the measures of oxidative damages in cell membranes, were significantly lower in OE line than that in the other lines under salt and osmotic stresses (Fig. 8c and d). Clearly, these findings indicated that overexpression of *ZmGPDH1* alleviated the cellular ROS accumulation by improving antioxidant defense and consequently minimize the cell death as well as membrane lipid peroxidation under salt and osmotic stresses.

### *ZmGPDH1* is involved in stress-induced stomatal closure

**Fig. 9:**
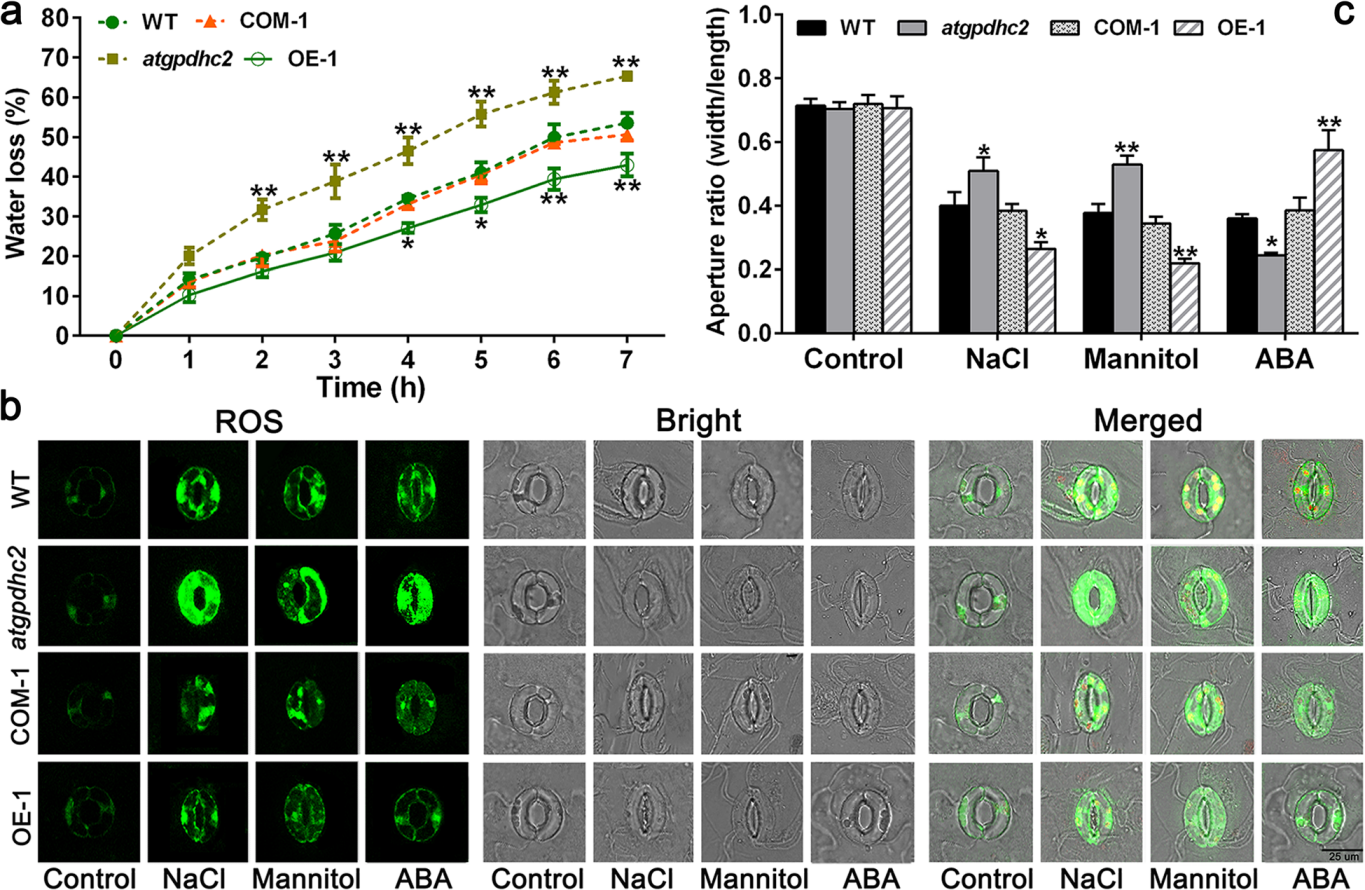
Overexpression of *ZmGPDH1* promotes the stomatal closure under salt and osmotic stresses. **a** The analysis of water loss rate (WLR) of the WT, *atgpdhc2*, COM-1 and OE-1. **b** For stomatal closure assays, the abaxial epidermis of rosette leaves were incubated in the light for 1 h to induce the stomatal opening and then treated with water (control), 300 mM mannitol, 150 mM NaCl and 20 μM ABA for 3 h. Bars = 25 μm. **c** Stomatal aperture was investigated by measuring the length and width of guard cells. Asterisks indicated significant differences from the WT, as determined by Student′s t-test (*P < 0.05, ** *P* < 0.01)

The water loss measurement revealed that overexpression of *ZmGPDH1* remarkably enhanced the transgenic plants resistance to water deficit, as reflected by a lower WLR (Fig. 9a). Since water loss mainly depends on the stomatal regulation, we further valuated whether *ZmGPDH1* was involved in the modulation of the stomatal closure under stress treatments. There was no significant difference in guard cells size or stomatal aperture ratio among WT, *atgpahc2*, COM-1 and OE-1 lines prior to stress treatment (Fig. 9b). By contrast, the NaCl or mannitol treatment markedly induced the stomatal closure in all the lines; nevertheless, the stomatal aperture ratio was dramatically lower in OE-1 plants and higher in *atgpdhc2* mutants, compared with WT plants. In addition, the stomatal aperture in COM-1 plants was generally similar with that in the WT (Fig. 9c). This implied that *ZmGPDH1* played an essential role in regulating the stomatal response to salt/osmotic stress.

The phytohormone ABA has the ability to induce stomatal closure [25]; however, overexpression of *ZmGPDH1* led to ABA insensitivity in stomatal movement (Fig. 9b). In the presence of ABA, the stomatal closure was significantly triggered in *atgpdhc2* mutants, moderately triggered in WT or COM and mildly triggered in OE plants, illustrating that *ZmGPDH1*-mediated stomatal closure was independent of ABA (Fig. 9c). In addition, the H_2_O_2_ production in the guard cells was also determined by H2DCF-DA staining. As shown in Fig. 9b, the H_2_O_2_ accumulation was less in OE plants and more in *atgpdhc2* mutant compared with that in the WT, suggesting that overexpression of *ZmGPDH1* contributed to sustain the ROS levels during stomatal movements.

### Effects of *ZmGPDH1* on expression of genes involved in redox homeostasis and ROS-scavenging system

**Fig. 10:**
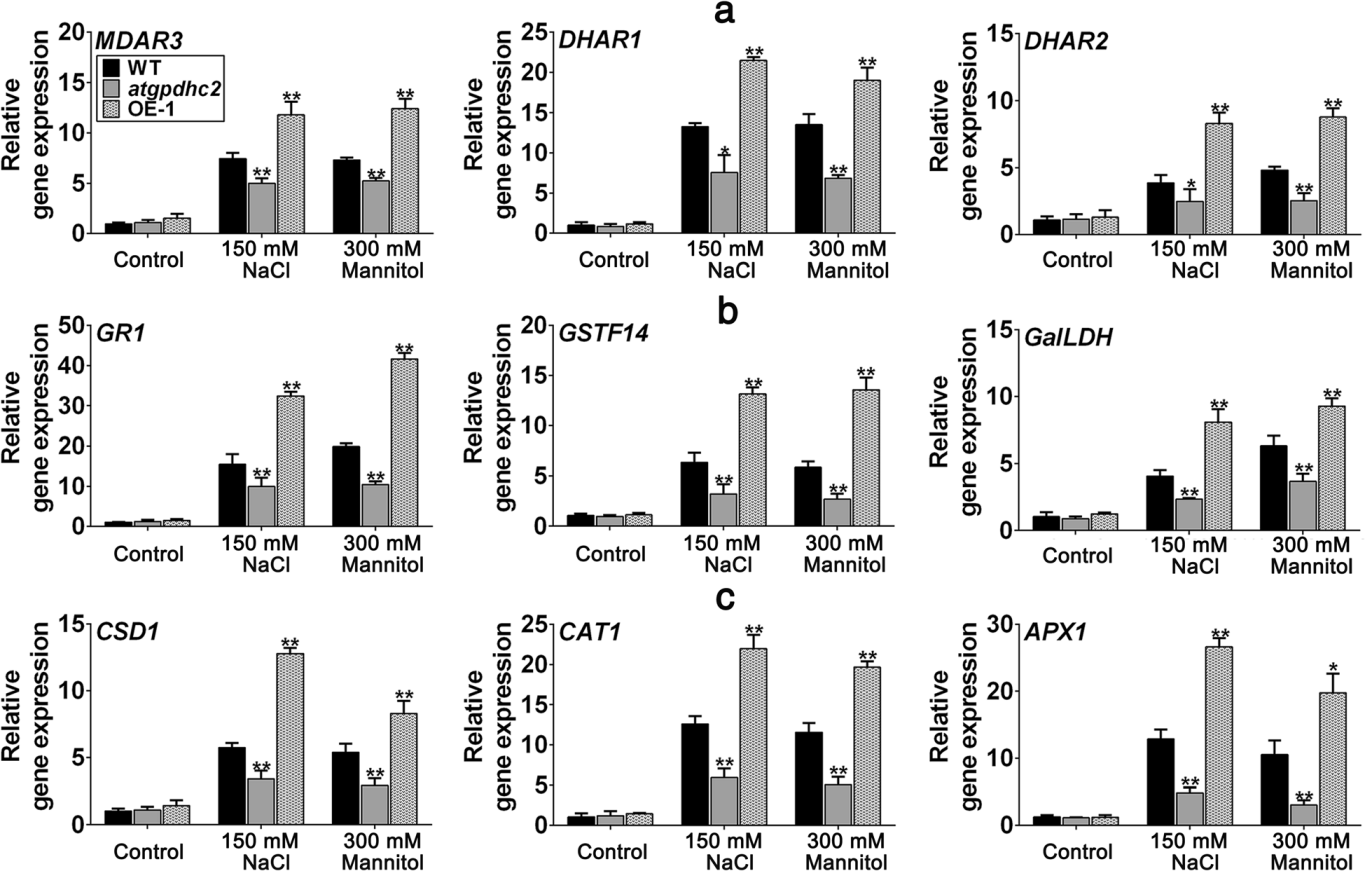
Overexpression of *ZmGPDH1* increases the transcripts of genes involved in cellular redox and ROS homeostasis. **a** and **b** Transcripts of genes involved in redox homeostasis (*MDAR3*, *DHAR1*, *DHAR2*, *GR1*, *GSTF14* and *GaILDH*). **c** Transcripts of genes involved in ROS-scavenging system (*CSD1*, *CAT1* and *APX1*). The roots of 3-week-old WT, *atgpdhc2*, and OE lines were submerged with water (control), 300 mM mannitol or 150 mM NaCl solution for 24 h, respectively. The expression of each gene in the WT treated with water was used to normalize its transcripts in different lines under different conditions. Asterisks indicated significant differences from the WT by Student′s t-test (*P < 0.05; **P < 0.01)

Next, to elucidate the impact of *ZmGPDH1* on molecular basis of salinity or osmotic stress response of cellular redox and ROS homeostasis, we detected the transcripts of a number of key genes involved in (1) ASA and GSH metabolism: cytosolic monodehydro-ascorbate reductase (*MDAR3*), cytosolic glutathione reductase (*GR1*), cytosolic dehydroascorbate reductase (*DHAR1*, *DHAR2*), cytosolic glutathione transferase (*GSTF14*) and cytosolic L-galactose dehydrogenase (*GalLDH*) [26–28] (2) ROS-scavenging system: cytosolic copper/zinc superoxide dismutase (*CSD1*), cytosolic catalase (*CAT1*) and cytoplasmic ascorbate peroxidase (*APX1*) [29, 30]. As shown in Fig. 10, the transcripts of all genes tested in *atgpdhc2* and OE-1 lines were generally similar to WT under normal conditions. Upon salinity or osmotic treatment, the transcripts of genes participating in ASA-GSH redox cycle (*MDAR3*, *GR1*, *DHAR1*, *DHAR2*) were decreased in *atgpdhc2* mutant but elevated in *ZmGPDH1* OE lines compared with the WT (Fig. 10a). Similarly, the transcripts of genes related to the biosynthesis of ASA and GSH (*GSTF14*, *GalLDH*) were also significantly increased in OE plants, despite which is not the case in *atgpdhc2* mutants. In addition, the transcripts of *CSD1*, *CAT1* and *APX1* were highly stimulated by NaCl or mannitol treatment, and were higher in *ZmGPDH1* OE line but lower in the *atgpdhc2* compare to the WT, indicating that overexpression of *ZmGPDH1* up-regulated the expression of the cytosolic antioxidant-related genes under both salt and osmotic stresses (Fig. 10b). These data also supported the finding that antioxidant activities of APX, SOD and CAT increased in the *ZmGPDH1* OE transgenic plants. Taken together, these results indicated that *ZmGPDH1* might function in the regulation of cellular redox and ROS homeostasis to prevent the oxidative damage caused by salt or osmotic stress.

## Discussion

Maize (*Zea mays* L.) is an important cereal crops as well as a major source of biofuel, industrial material and animal feed [31]. Although a great deal of research has indicated that glycerol-3-phosphate dehydrogenase (GPDH) plays a pivotal role in plant growth and stress adaptions [3, 17, 21], little is currently known about its functions in field crops including maize. In this study, we isolated a *GPDH* gene encoding NAD^+^-dependent GPDH from maize. Similar to other typical NAD^+^-dependent GPDH [14, 16, 19], ZmGPDH1 protein contained the necessary and specific protein domains (PF07479, PF01210). The conserved GAGAWG motif was found at residues 44–50 of the protein sequence of *ZmGPDH1* (Additional file 1: Figure S1), which was similar to the previously reported NAD^+^-dependent GPDH isoforms with an analogous NAD^+^-binding fragments corresponding to GXGXXG [8, 10, 32].

Enzymatic assay of recombinant ZmGPDH1 proteins expressed in *Escherichia coli* Rosetta strain (DE3) (Fig. 1e and f) showed the purified ZmGPDH1 protein had substrate affinity (*K*_m_^DHAP^ of 2.75 mM) (Fig. 1e), which compared well with the kinetic parameters previously reported for other GPDH enzymes [10, 33, 34]. The stable or transient expression of a green fluorescent protein (GFP)-tagged *ZmGPDH1* in *Arabidopsis* or wild-type rice were conducted, and both evidenced that ZmGPDH1 proteins were specially targeted to cytosol (Additional file 2: Figure S2 and Fig. 1a). The earlier identified *Arabidopsis* GPDH proteins (AtGPDHc1 and AtGPDHc2) were predicted to be cytosol-located GPDs owing to the absence of apparent transmembrane regions and subcellular targeting sequences, however, a clear experimental evidence was lacking [5, 8].

In succession, the physiological functions of the cytosolic *ZmGPDH1* gene in mediating salinity/osmotic adaption were investigated in this study. The transcript abundance of *ZmGPDH1* was markedly increased under NaCl and mannitol treatments (Fig. 2n and o). On the other hand, the transgenic *Arabidopsis* harboring the *ZmGPDH1* promoter fused to a *GUS* reporter gene also showed relatively higher GUS activity under NaCl or mannitol condition (Fig. 2p), indicating that *ZmGPDH1* gene was regulated at the transcription level in response to salt or osmotic stress, which was consistent with the expression patterns of other *GPDH* genes from *A.thaliana* (*AtGPDHc1*, *AtGPDHm1*), *C. reinhardtii* (*CrGPDH2*, *CrGPDH3*), *D. salina* (*DsGPDH2*, *G3PDH*) and *D. viridis (DvGPDH1*, *DvGPDH2*) [6, 8, 9, 13, 19]. Furthermore, overexpression of *ZmGPDH1* strongly enhanced the tolerance of *Arabidopsis* (WT) to salt/osmotic stress and rescued the salt/osmotic sensitivity of *atgpdhc2* mutant, as reflected by a pronounced elevation in germination rate, fresh weight, root length, biomass, chlorophyll content and *F*_v_/*F*_m_ ratio under salinity or osmotic conditions (Figs. 3 and 4). Similar results have been reported earlier in other species: overexpression of an oyster mushroom *GPDH* gene (*PsGPD*) increased the salt tolerance in transgenic potatoes and rice; and overexpression of a black yeast *GPDH* gene (*HwGPD1B*) also enhanced NaCl tolerance of *Saccharomyces cerevisiae gpd1* mutant [17, 35].

We also found that *ZmGPDH1* gene was required for glycerol generation. Glycerol is an important osmo-protectant and its accumulation can compensate for differences between intracellular and extracellular water potentials under hyperosmotic environment [6, 12, 13]. In our study, overexpression of *ZmGPDH1* markedly increased the levels of G-3-P and glycerol, demonstrated that *ZmGPDH1* played essential roles in plant adaptation to hypersaline or hyperosmotic shock by contributing to the glycerol biosynthesis (Fig. 5). Likewise, of the five GPDH enzymes in *Chlamydomonas reinhardtii*, *CrGPDH2* and *CrGPDH3* were shown to be necessary for osmotic-induced glycerol production [36]. Also, loss of *AtGPDHc1* gene encoding a cytosol-localized GPDH of *Arabidopsis* caused the hypersensitivity to salt stress due to the severe impairment in provision of glycerol [8].

**Fig. 11:**
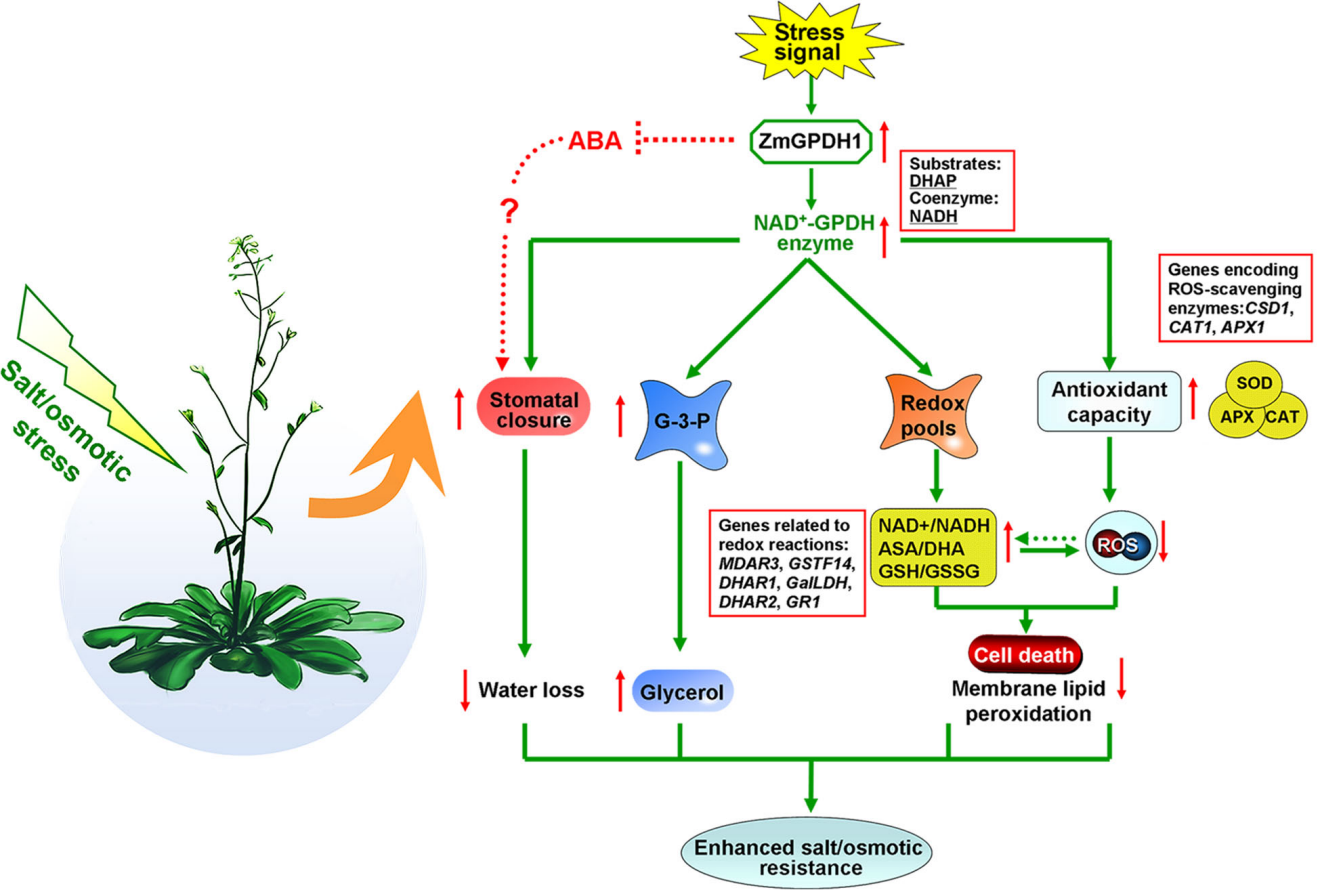
Model of the involvement of *ZmGPDH1* in salt/osmotic stress responses. The stress signals triggered the expression of *ZmGPDH1* and increased the NAD^+^-GPDH enzyme activity, which was required for two biological processes: glycerol biosynthesis by affecting the G-3-P provision and NADH/NAD^+^ homeostasis by disposing of extra reducing power. The elevated expression of genes involved in ROS and redox homeostasis resulted in improved ROS scavenging capacity and a series of positive physiological changes. In addition, the boosted accumulation of G-3-P/glycerol and optimized stomatal movement/water loss were also in concurrence, and collectively contributed to an enhanced salt/osmotic resistance. Red arrows indicated the tendency of changes

Most notably, cytosolic GPDHs are reported to participate in the mitochondrial G-3-P shuttle system, which functions as a pivotal route to keep the cellular redox status in *Arabidopsis* [8]. In this study, *ZmGPDH1* overexpression *Arabidopsis* showed significantly decreased NADH level accompanied by an increased NAD^+^ accumulation during salt/osmotic stress (Fig. 6a). As a consequence, a noticeable reduction in cellular NADH/NAD^+^ ratio was detected in OE lines, suggested that *ZmGPDH1* involved in the regulating of NADH/NAD^+^ redox homeostasis by consuming the excessive redundant NADH and regenerating NAD^+^ under salt or osmotic stress. In addition, an apparent increase in ASA and GSH contents as well as their redox pool were observed in *ZmGPDH1* overexpression *Arabidopsis* compared with that in WT plants (Fig. 6b and c), indicated that overexpression of *ZmGPDH1* also affected the redox states of ASA and GSH, apart from a decreased cellular NADH/NAD^+^ ratio. It is known that GR, MDHAR and DHAR are responsible for the regeneration of ASA and GSH in ASA-GSH redox cycle [37]. GalLDH and GST are proved to be essential enzymes involved in the biosynthesis of ASA and GSH, respectively [28]. In the present study, the increased expression of the marker genes corresponding to the above mentioned enzymes in the *ZmGPDH1*-OE plants implied that *ZmGPDH1* might exert effects on the ASA/GSH redox cycles as well, and play a critical role in optimizing the cellular redox homeostasis of NADH/NAD^+^, ASA/DHA and GSH/GSSG under salt and osmotic stresses, as speculated in Fig. 11. Overexpression of *ZmGPDH1* also caused a reduction in ROS level, including the guard cell ROS, as exhibited by the slight H2DCF-DA staining under stress treatments (Fig. 7 and Fig. 9). Besides, the level of lipid peroxidation and cell death in *ZmGPDH1* OE lines was much lower than that of the other plants (Fig. 8), illustrating that overexpression of *ZmGPDH1* availably protected cell from oxidative damage and maintained the membrane integrity under salt or osmotic stress. It has also been reported that the soluble redox couples seem to assume a dual role with respect to ROS [28]. On one hand, the excessive NADH can be involved in, or related to ROS-generation metabolic pathway by triggering the over-reduction of molecular oxygen O_2_ [38]. In contrast, the reductive detoxification of ROS also heavily depends on the NADH oxidation, as signified by the increased NAD^+^/NADH ratio, which helps to enhance the oxidant scavenging capacity [23, 28]. Hence, it appeared that *ZmGPDH1* might participate in the regulation of ROS metabolism by manipulating the cellular NADH/NAD^+^ ratio (Fig. 11). On the other hand, the antioxidant system has been proved to be dramatically evoked to eliminate excess ROS under abiotic stress [39]. In agreement with this, we found that the activities and transcripts of ROS-scavenging enzymes (*CSD1*, *CAT1*, and *APX1*) were strongly stimulated by salinity or mannitol in OE or COM plants (Fig. 7 and Fig. 10). In summary, the overexpression of *ZmGPDH1* resulted in higher expression of genes encoding key enzymes of cytosolic ROS scavenging system involving the SOD/CAT/ascorbate/ glutathione cycle, which led to lower ROS accumulation and higher ROS detoxification capacity, and hence stronger stress tolerance (Fig. 11).

Additionally, our results showed that *ZmGPDH1*-OE plants exhibited greater stomatal closure compared with other lines, indicating that *ZmGPDH1* played a key role in manipulating the stomatal closure under salt or osmotic stress (Fig. 9). However, the ABA-induced stomatal closing was impaired in *ZmGPDH1*-OE lines but significantly induced in *atgpdhc2* mutant, suggesting that the *ZmGPDH1*-mediated stomatal closure might be independent of ABA. Meanwhile, we found that overexpression of *ZmGPDH1* reduced the plant sensitivity to ABA at seed germination and early seedling developmental stages (Additional file 5: Figure S5), which was also observed previously on *AtGPDHc1* mutants [8]. Further studies will be needed to interpret the interaction of *ZmGPDH1* and ABA signaling pathway.

## Conclusions

We reported the characterization of a cytosolic NAD^+^-dependent GPDH gene from maize, *ZmGPDH1*, which had profound effects on salt/osmotic tolerance by regulating the glycerol accumulation, cellular redox homeostasis, ROS-scavenging system as well as stomatal movement (Fig. 11).

## Methods

### Plant materials and growth conditions

The maize inbred line accession He-344 (provided by Heilongjiang Academy of Agricultural Sciences, Harbin, China) was used as the plant material in this experiment and normally planted in a growth chamber under controlled photoperiod and temperature (12 h light/12 h dark, 23 ± 2 °C), with a photon flux density of 1000 μmol m^− 2^ s^− 1^.

The seeds of T-DNA insertion mutants of *AtGPDHc2* (TAIR: At3G07690), namely *atgpdhc2* (SALK_033040) were donated by Dr. Pradeep Kachroo (University of Kentucky, USA). The homozygous lines of *atgpdhc2* mutant were identified by PCR and reverse transcription PCR (RT-PCR) analysis, and the primers were shown in Additional file 6: Table S1. The plants of *Arabidopsis thaliana* (ecotype Col) and *atgpdhc2* mutant (ecotype Col) were grown in a growth chamber under controlled photoperiod and temperature (16 h light/8 h dark, 21 ± 2 °C), with a photon flux density of 100 μmol m^− 2^ s^− 1^.

### Plasmid construction and plant transformation

The full length coding region of *ZmGPDH1* (1377 bp) was cloned from cultivated maize by the gene specific primers (Additional file 6: Table S1). The PCR product was purified and inserted into the *Xba*I and *Sal*I sites of the pBI121-GFP vector under control of the CaMV35S promoter. For complementation and over-expression assays, *Agrobacterium tumefaciens* strain EHA105 carrying the construct *pBI121-ZmGPDH1::GFP* was used to transform *Arabidopsis* wild-type or *atgpdhc2*. T3 homozygous transgenic *Arabidopsis* were screened by RT-PCR.

### Protein subcellular localization and GUS activity assay

To verify the subcellular localization of *ZmGPDH1*, the mesophyll protoplasts were isolated from T3 homozygous transgenic *Arabidopsis* harboring *pBI121*-*ZmGPDH1::GFP* or *pBI121*-GFP (control) plasmid, and the subcellular localization of GFP expression was visualized by confocal laser-scanning microscope (Leica, German). The positive control (empty vector) or fusion proteins were also temporarily expressed in rice mesophyll protoplasts according to the methods described previously [40]. For co-localization studies, a far-red fluorescent protein mkate was used as the cytosol marker. To study the promoter activity, a 1642-bp genomic region upstream of the translation initiation codon of *ZmGPDH1* gene was cloned into pBI121-GUS at *Hind*III and *Xba*I sites (primers see Additional file 1: Table S1). The constitutive *proZmGPDH1::GUS* transformed WT plants were also generated and T3 homozygous transgenic lines were used for GUS staining according to the reported protocol [41]. The images were visualized by stereo microscope (Olympus, Japan).

### Recombinant ZmGPDH1 protein expression, purification and western blot

The coding region of *ZmGPDH1* with the *Nco*I and *Xho*I sites was amplified by PCR and then inserted into the pET32a (+) vector containing 6 × His tag. The *pET32a-ZmGPDH1* plasmid was transformed into the *Escherichia coli* (*E. coli*) Rosetta strain and the expression of *ZmGPDH1* was induced with 1 mM IPTG to generate the putative recombinants. Then the His-tagged ZmGPDH1 proteins were extracted and purified under native conditions using Ni-NTA nickel columns (Sigma), and the purified proteins were detected by 12% SDS-PAGE as well as Western blot using 6 × His Tag Antibody as probe.

### Phenotypic analyses of the *ZmGPDH1* transgenic *Arabidopsis* under salt or osmotic treatment

For germination analysis, seeds of WT, *atgpdhc2*, OE and COM lines were plated on half-strength MS plates containing 200 mM mannitol or 100 mM NaCl for 8 days. Germination rates were counted at day 5 after sowing and seed germination was defined as the appearance of visible radicle. To investigate the effects of salinity and osmotic stresses on root length and fresh weight, 7-days-old seedlings of WT, *atgpdhc2* mutant, COM and OE lines were transferred into half-strength MS plates supplemented with 150 mM NaCl or 300 mM mannitol. The root length and fresh weights of stress-treated seedlings were determined after 7 day of treatment.

For the stress tolerance test at the adult stage, 3-week-old *Arabidopsis* plants were irrigated with 200 mM NaCl or 400 mM mannitol solution every 3 days for a total of 9 days. Rosette leaf samples were collected at day 6 of treatments to measure the changes of various physiological and biochemical parameters. All experiments were replicated at least three times with 80–100 plants per treatment. Photographs taken from one representative experiment are shown. Total chlorophyll (chlorophyll a + b) was determined according to the method as previously described [42] and the fresh young leaf was extracted in 80% (*v*/v) acetone extract. Photochemical efficiency (*F*_*v*_/*F*_*m*_) was examined by using a pulse*-*modulated fluorometer (FMS2, Hansatech, UK) [43].

To investigate the water loss rate (WLR), the rosette leaves from 4-week-old *Arabidopsis* were weighed at specific time points. The decrease in fresh weight was used to calculate WLR. For stomatal closure assays, the strips abaxial epidermis of *Arabidopsis* leaves were immerged in buffer (10 mM MES/KOH, pH 6.1, 10 mM KCl, 50 μM CaCl_2_) under light for 1 h to induce the stomatal opening and then treated with 300 mM mannitol, 150 mM NaCl and 20 μM ABA for 3 h. The conformation of stomatal aperture were photographed by confocal microscope and processed with ImageJ software. The experiments were replicated at least three times with 40–50 cells per treatment.

### Analysis of GPDH activity, G-3-P and glycerol levels

G-3-P and glycerol contents were measured as previously described with slight modifications [44]. The GPDH activity was examined with regard to the reduction of DHAP by NADH. The total reaction volume of the assay was 1 mL containing 100 mM HEPES buffer, pH 6.9, 4 mM DHAP, 0.2 mM NADH and an appropriate amount of enzyme [8]. The absorbance changes at 340 nm were monitored using an ultraviolet spectrophotometer (U3900, Hitachi High-Technologies, Japan).

### Analysis of cellular redox and ROS homeostasis

The reduced pyridine nucleotides (NADH) content, oxidized pyridine nucleotides (NAD^+^) content and NADH/NAD^+^ ratio were assayed with an enzymatic cycling procedure [45]. The ascorbate (ASA) content, dehydroascorbate (DHA) content and ASA/DHA ratio were measured following the reported protocols [46]. The glutathione (GSH) content, oxidized glutathione (GSSG) content and GSH/GSSG ratio were assayed as described [47].

For ROS accumulation analysis, the staining of nitroblue tetrazolium (NBT) and 3,3- diaminobenzidine (DAB) of stress-treated seedlings were performed following the reported protocol [48]. For hydrogen peroxide (H_2_O_2_) staining in the guard cells, prepared epidermal peels with NaCl, mannitol or ABA treatment were stained with 2,7-dichlorofluorescin diacetate (H2DCF-DA) for 10 min [49]. Cell death caused by salt or osmotic stress was also estimated by Evan′s blue and PI staining as described [24]. The assays of H_2_O_2_ and superoxide (O_2_^.-^) were conducted by spectrophotometry as previously described [50, 51]. The lipid peroxidation was measured with reference to the thiobarbituric acid-reactive substances (TBARS) content [52]. Electrolyte leakage (EL) was assessed as described [53].

To monitor the antioxidant enzyme activities, leaf tissues (0.5 g) were ground in ice bath with 10 mL extraction buffer (K_2_HPO_4_-KH_2_PO_4_, pH 7.0, 1.5 mM EDTA, 1% PVP, 0.5 mM ASC), and then the homogenate was centrifuged at 12000 rpm for 20 min at 4 °C. The supernatant was used for the determination of enzymes activities. The activities of catalase (CAT), ascorbate peroxidase (APX) and superoxide dismutase (SOD) were determined as described [54, 55], with slight modifications.

### Quantitative real-time RT-PCR analysis

To analyze the expression of *ZmGPDH1* under osmotic and salt stresses, 3-week-old maize seedlings were treated with 1/2 Hoagland solution containing 400 mM mannitol and 200 mM NaCl solutions for 0, 1, 3, 6, 12 and 24 h, and the roots were sampled to analyze the transcripts of *ZmGPDH1*. The untreated maize samples from the same time point were used as the controls. *ZmGAPDH* and *ZmACTIN* genes served as internal reference in each assay. To examine the tissue-specific expression of *ZmGPDH1*, total RNA was extracted from rosette leaves (RL), flower buds (FLB), roots (RT), flowers (FL), siliques (SL), stems (ST) and cauline leaves (CL) in *proZmGPDH1::GUS* transgenic plants. To analyze target genes expression induced by osmotic and salt stresses, 3-week-old WT, *atgpdhc2*, and OE lines were treated with water (control), 300 mM mannitol or 150 mM NaCl solution. Total RNA was extracted from rosette leaf samples at 24 h after treatments. The expression of target genes in WT plants under control environment was used as a calibrator. *ACTIN2* and *UBQ7* genes were used as internal reference [56]. The primers used for transcriptional analysis were shown in Additional file 6: Table S1.

### Statistical analysis

Data are presented as Mean ± SD. The Student’s t-test was used to determine the significance levels using SPSS 21.0 software throughout this study. A *P*-value of < 0.05 was considered statistically significant.

## Additional files

Additional file 1: **Figure S1.** Alignment analysis of the *ZmGPDH1* and *AtGPDHc2* protein sequence (TIF 911 kb)

Additional file 2: **Figure S2.** Subcellular localization of pBI121-ZmGPDH1::GFP fusion proteins in rice mesophyll protoplasts. **a** Confocal micrographs showing localization of GFP and ZmGPDH1-GFP. **b** Confocal micrographs showing localization of ZmGPDH1-GFP in mesophyll protoplasts expressing a far-red fluorescent protein mkate (TIF 2244 kb)

Additional file 3: **Figure S3.** Kinetic analysis of the GPDH activity of ZmGPDH1. (TIF 43 kb)

Additional file 4: **Figure S4.** Molecular characterization of the *atgpdhc2* mutant. a Genomic organization of the *atgpdhc2* location. b Identification of homozygous mutants. M: DL2000 marker; LP and RP: Forward and reverse primers of target genes; LB: The T-DNA left border primer. **c** Reverse transcription PCR (RT-PCR) of *AtGPDHc2* transcripts in *atgpdhc2* mutants and wild-type (WT) *Arabidopsis*. (TIF 1762 kb)

Additional file 5: **Figure S5.** Phenotype of *ZmGPDH1* OE lines in response to ABA. a The seeds of WT and OE lines were germinated on half-strength MS plates with or without ABA. b Germination rate of WT and OE lines under different concentrations of ABA treatment at day 5 after imbibitions. c 7-day-old WT and OE seedlings were grown on half-strength MS plates without or with ABA for 7 days. d The fresh weigh and primary root length of WT and OE seedlings after ABA treatment. Asterisks indicate significant differences from WT plants by Student′s t-test (*P < 0.05; **P < 0.01). (TIF 10675 kb)

Additional file 6: **Table S1.** The gene ID and primers used in this study. (PDF 86 kb)

## Abbreviations

ABA: Abscisic acid
APX: Ascorbate peroxidase
ASA: Ascorbate
CAT: Catalase
CL: Cauline leaves
DAB: 3,3-diaminobenzidine
DHA: Dehydroascorbate
DHAP: Dihydroxyacetone phosphate
EL: Electrolyte leakage
FL: Flowers
FLB: Flower buds
G-3-P: Glycerol-3-phosphate
GFP: Green fluorescent protein
GK: Glycerol kinase
GPDH: Glycerol-3-phosphate dehydrogenase
GSH: Glutathione
GSSG: Oxidized glutathione
H2DCF-DA: 2,7-dichlorofluorescin diacetate
H_2_O_2_: Hydrogen peroxide
NAD^+^: Oxidized pyridine nucleotides
NADH: Reduced pyridine nucleotides
NBT: Nitroblue tetrazolium
O_2_^.-^: Superoxide
PI: Propidium iodide
qRT-PCR: quantitative real-time PCR
RL: Rosette leaves
RT: Roots
RT-PCR: reverse transcription PCR
SL: Siliques
SOD: Superoxide dismutase
ST: Stems
TBARS: Thiobarbituric acid-reactive substances
WLR: Water loss rate
WT: Wild-type
