# Supplementary figures and images for: A cytosolic NAD+-dependent GPDH from maize (ZmGPDH1) is involved in conferring salt and osmotic stress tolerance
Source: BMC Plant Biol. 2019 Jan 9;19:16. doi: 10.1186/s12870-018-1597-6 (PMC6327487; doi:10.1186/s12870-018-1597-6)

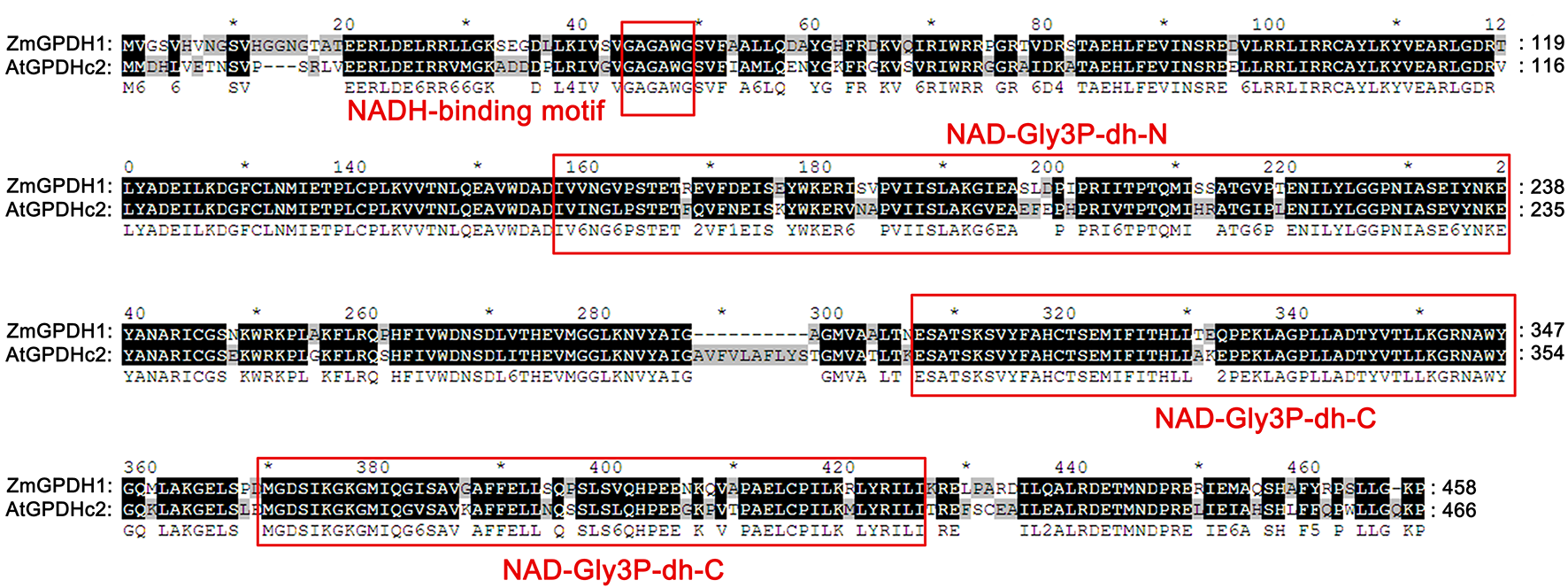

Supplement: Supplementary file 1 — Figure S1. Alignment analysis of the ZmGPDH1 and AtGPDHc2 protein sequence (TIF 911 kb) [file 12870_2018_1597_MOESM1_ESM.tif]

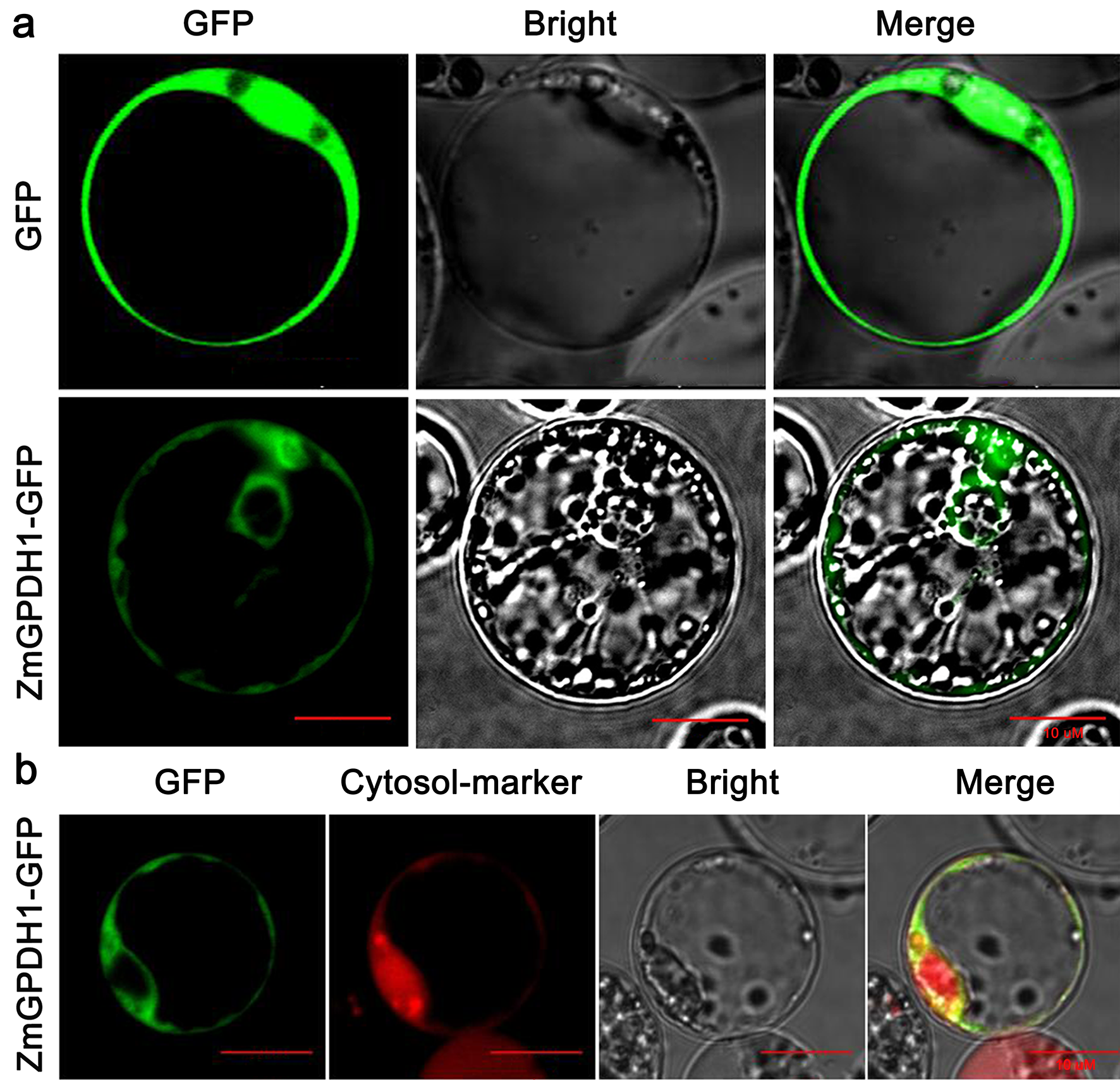

Supplement: Supplementary file 2 — Figure S2. Subcellular localization of pBI121-ZmGPDH1::GFP fusion proteins in rice mesophyll protoplasts. a Confocal micrographs showing localization of GFP and ZmGPDH1-GFP. b Confocal micrographs showing localization of ZmGPDH1-GFP in mesophyll protoplasts expressing a far-red fluorescent protein mkate (TIF 2244 kb) [file 12870_2018_1597_MOESM2_ESM.tif]

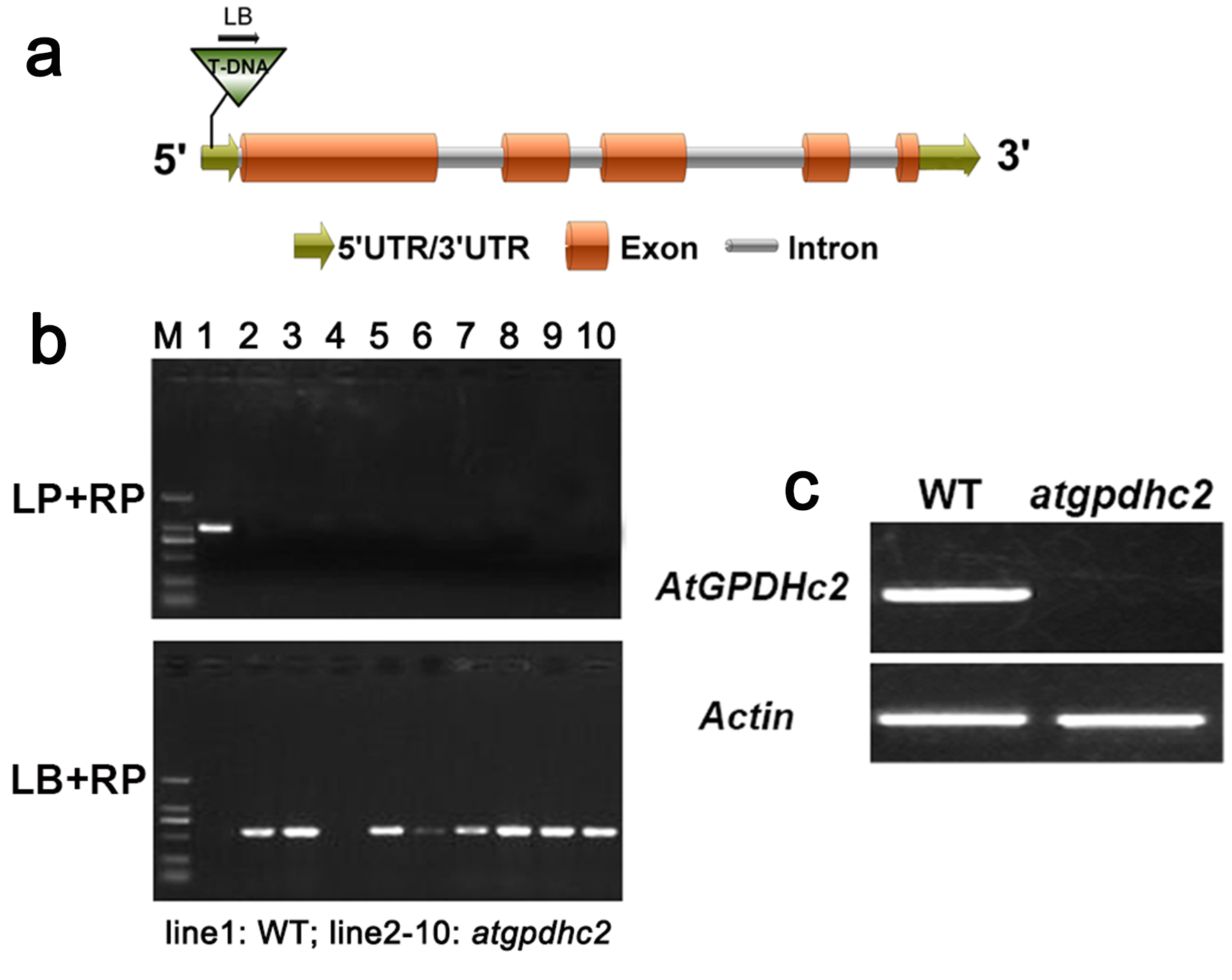

Supplement: Supplementary file 4 — Figure S4. Molecular characterization of the atgpdhc2 mutant. a Genomic organization of the atgpdhc2 location. b Identification of homozygous mutants. M: DL2000 marker; LP and RP: Forward and reverse primers of target genes; LB: The T-DNA left border primer. c Reverse transcription PCR (RT-PCR) of AtGPDHc2 transcripts in atgpdhc2 mutants and wild-type (WT) Arabidopsis. (TIF 1762 kb) [file 12870_2018_1597_MOESM4_ESM.tif]

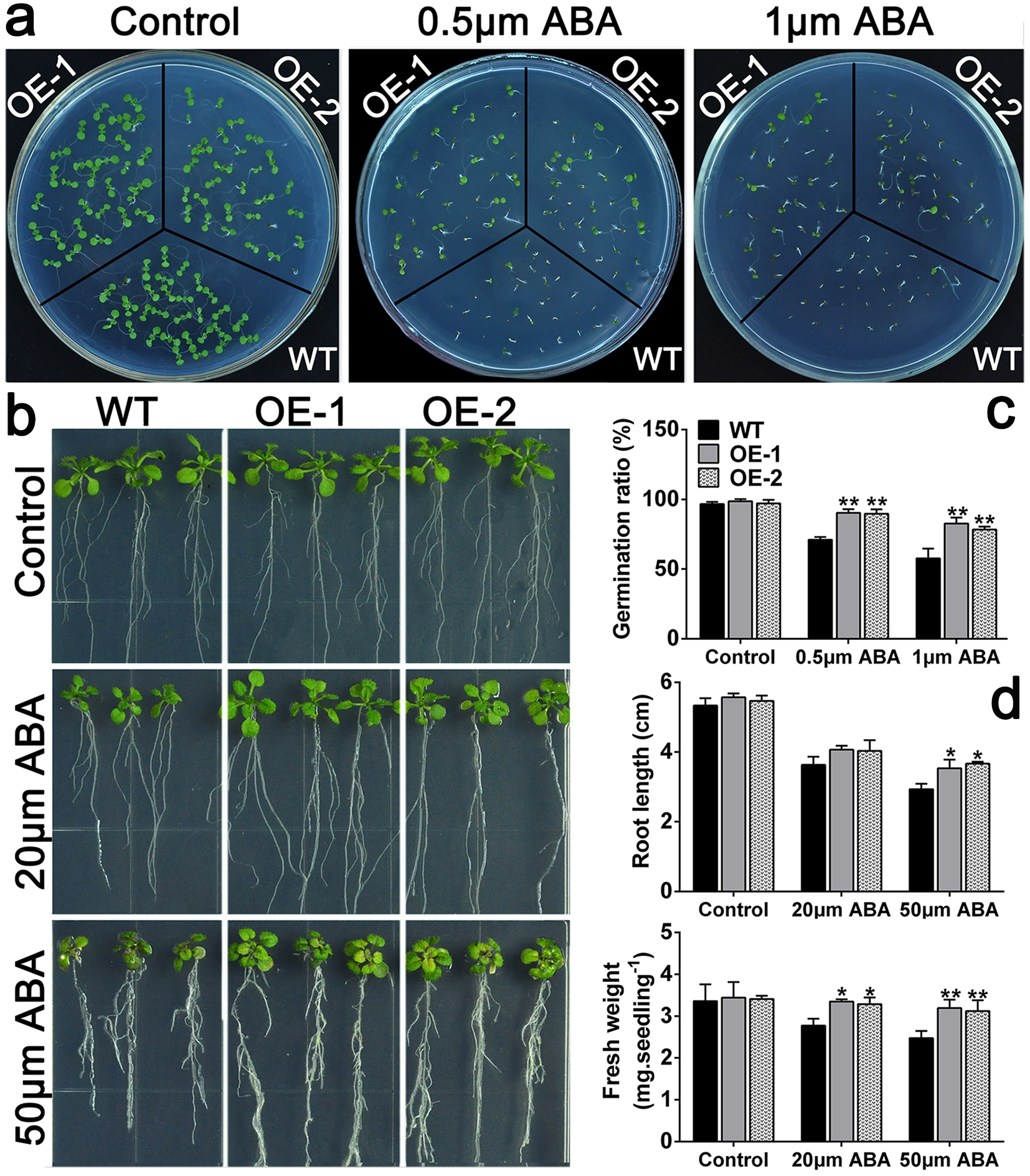

Supplement: Supplementary file 5 — Figure S5. Phenotype of ZmGPDH1 OE lines in response to ABA. a The seeds of WT and OE lines were germinated on half-strength MS plates with or without ABA. b Germination rate of WT and OE lines under different concentrations of ABA treatment at day 5 after imbibitions. c 7-day-old WT and OE seedlings were grown on half-strength MS plates without or with ABA for 7 days. d The fresh weigh and primary root length of WT and OE seedlings after ABA treatment. Asterisks indicate significant differences from WT plants by Student′s t-test (*P < 0.05; **P < 0.01). (TIF 10675 kb) [file 12870_2018_1597_MOESM5_ESM.tif]
