# Supplementary material for: A cytosolic NAD+-dependent GPDH from maize (ZmGPDH1) is involved in conferring salt and osmotic stress tolerance
Source: BMC Plant Biol. 2019 Jan 9;19:16. doi: 10.1186/s12870-018-1597-6 (PMC6327487; doi:10.1186/s12870-018-1597-6)
Supplement: Supplementary file 6 — Table S1. The gene ID and primers used in this study. (PDF 86 kb) [file 12870_2018_1597_MOESM6_ESM.pdf]

Table S1. The gene ID and primers used in this study.

Primer Name	Sequence (5'-3')	Gene ID	Description
AtGPDHc2	LP-GAGATGGCTGTGAGCAATCTC<br>RP-ACCTCCTCAACAATTCTTCCC<br>LB-GCGTGGACCGCTTGCTGCAACT	TAIR:AT3G07690	Mutant identification
ZmGPDH1	Forward: ATGGTTGGGAGCGTGCACGTC<br>Reverse: TCATGGTTTTCCAAGGAGAGACG	GeneBank:MH460963	cloning
ProZmGPDH1	Forward: CAAT CGGCCGTCAGCATTC<br>Reverse: TTCCCGAGCTCAGGAGTCAG	GeneBank:MH483980	cloning
AtGPDHc2	Forward: GAGTTTGAGCCTCATCCTC<br>Reverse: CATTAGGTGGTCGGTGAGGT	TAIR:AT3G07690	RT-PCR
ZmGPDH1	Forward: AAGGGAGAGTTGAGTCCTG<br>Reverse: AGTATTCTGT AAAGCCTC	GeneBank:MH460963	qRT-PCR
GUS	Forward: GTCGCGCAAGACTGTAACCA<br>Reverse: TGGTTAATCAGGAACTGTTG	GeneBank:KM434773	qRT-PCR
GSTF14	Forward: ATCTGGGGTAATTCGGCAGC<br>Reverse: TTTTGGCTTCACCAGCAAGC	TAIR:AT1G49860	qRT-PCR
MDAR3	Forward: ACAAAGAGAAAGAGATAGTCAAAGC<br>Reverse: TGCTTCTTGGACGCCAATTTC	TAIR:AT3G09940	qRT-PCR
CSD1	Forward: TGAACTCAGCCTGGCTACTGG<br>Reverse: AGCCACACACCAGAAGATACACAC	TAIR:AT1G08830	qRT-PCR
CAT1	Forward: TGGGATTCAGACAGGCAAGAACG<br>Reverse: GTTTGGCCTCACGTTAAGACGAGT	TAIR:AT1G20630	qRT-PCR
APX1	Forward: TTTCCACCCTGGAAGAGAGGAC<br>Reverse: TCACAACCCTTGGTAGCATCAGG	TAIR:AT1G07890	qRT-PCR
GR1	Forward: ATTCGTGGCAGATGTCGT<br>Reverse: CACAGCTCCAGCCTGATC	TAIR:AT3G24170	qRT-PCR
DHAR1	Forward: TGGCTCTGGAAATCTGTG<br>Reverse: AGCAAGGCATGTTCAGATCC	TAIR:AT3G24170	qRT-PCR
DHAR2	Forward: AATGGGTGGCTGATTCTGAC<br>Reverse: CCGCAACCACAATCTCTTTC	TAIR:AT1G75270	qRT-PCR
GalDH	Forward: AGTCGCCGAAGATGATGCCGT<br>Reverse: GACAATATCACATCGACAGT	TAIR:AT4G33670	qRT-PCR
ACTIN2	Forward: GCTGACCGTATGAGCAAAGA<br>Reverse: GATCCACATCTGTTGGAACG	TAIR:AT3G18780	qRT-PCR
UBQ10	Forward: GGCCTTGTATAATCCCTGATGAA<br>Reverse: AGAAGTCGACTTGTCATTAGAAAGAAA	TAIR:AT4G05320	qRT-PCR
ZmGAPDH	Forward: CCCTTCATCACCACGGACTAC<br>Reverse: AACCTTCTTGGCACCACCCT	GeneBank:XM_020551757.1	qRT-PCR
ZmACTIN	Forward: ATCCAGGCTGTTCTTTCGTT<br>Reverse: CATTAGGTGGTCGGTGAGGT	GeneBank:XM_008656735.2	qRT-PCR
